# BRD-810 is a highly selective MCL1 inhibitor with optimized in vivo clearance and robust efficacy in solid and hematological tumor models

**DOI:** 10.1038/s43018-024-00814-0

**Published:** 2024-08-23

**Authors:** Ulrike Rauh, Guo Wei, Michael Serrano-Wu, Georgios Kosmidis, Stefan Kaulfuss, Franziska Siegel, Kai Thede, James McFarland, Christopher T. Lemke, Nicolas Werbeck, Katrin Nowak-Reppel, Sabine Pilari, Stephan Menz, Matthias Ocker, Weiqun Zhang, Kyle Davis, Guillaume Poncet-Montange, Jennifer Roth, Douglas Daniels, Virendar K. Kaushik, Brian Hubbard, Karl Ziegelbauer, Todd R. Golub

**Affiliations:** 1 Trueline Therapeutics Inc., Cambridge, MA USA; 2 Broad Institute of MIT and Harvard, Cambridge, MA USA; 3 Anji Pharmaceuticals Inc., Cambridge, MA USA; 4 Ncardia, Leiden, The Netherlands; 5 Nuvisan Innovation Campus Berlin, Berlin, Germany; 6 Bayer AG R&D, Berlin, Germany; 7 Independent Consultant, Pharmacometrics Modeling and Simulation, Berlin, Germany; 8 Almirall R&D, Sant Feliu de Llobregat, Spain; 9 Department of Pediatric Oncology, Dana-Farber Cancer Institute and Harvard Medical School, Boston, MA USA

**Keywords:** Cancer, Drug development, Apoptosis

## Abstract

The *MCL1* gene is frequently amplified in cancer and codes for the antiapoptotic protein myeloid cell leukemia 1 (MCL1), which confers resistance to the current standard of care. Therefore, MCL1 is an attractive anticancer target. Here we describe BRD-810 as a potent and selective MCL1 inhibitor and its key design principle of rapid systemic clearance to potentially minimize area under the curve-driven toxicities associated with MCL1 inhibition. BRD-810 induced rapid cell killing within 4 h in vitro but, in the same 4-h window, had no impact on cell viability or troponin I release in human induced pluripotent stem cell-derived cardiomyocytes, even at suprapharmacologic concentrations*.* In vivo BRD-810 induced efficacy in xenograft hematological and solid tumor models despite the short residence time of BRD-810 in plasma. In totality, our data support the hypothesis that short-term inhibition of MCL1 with BRD-810 can induce apoptosis in tumor cells while maintaining an acceptable safety profile. We, therefore, intend to advance BRD-810 to clinical trials.

## Abstract (web summary)

Rauh et al. developed a selective MCL1 inhibitor that is efficacious in hematological and solid tumors and has the advantage of limited cardiotoxicity because of more rapid clearance of the drug in vivo.

## Main

Resistance to programmed cell death, termed apoptosis, is a hallmark of cancer^1^. Every cell is equipped to sense extrinsic and intrinsic stress signals and maintain a proper balance of proapoptotic and antiapoptotic factors, which ultimately determines the life or death of the cell. In malignant cells, the B cell lymphoma 2 (Bcl-2) protein family has a central role and myeloid cell leukemia 1 (MCL1), as one of the key antiapoptotic factors of that protein family, has been identified to promote aberrant cell survival^2^.

Like other antiapoptotic Bcl-2 family members, MCL1 prevents cancer cell death by tightly binding to proapoptotic effector proteins including BAK, BAX or BOK, as well as BH3-only activator proteins such as BIM, PUMA or NOXA^3–7^. Unbound proapoptotic effector proteins initiate the cell death cascade by forming pores in the mitochondrial outer membrane, leading to a breakdown of the mitochondrial outer membrane potential and release of Ca^2+^ and cytochrome *c* to the cytosol where the apoptosome is formed. This triggers an irreversible cascade of caspase activation and enzymatic autoproteolysis, ultimately leading to cell death^8,9^.

MCL1 has distinguishing features compared to other antiapoptotic Bcl-2 proteins. MCL1 has been shown to interact with BOK through its C-terminal transmembrane domain and somatic mutations, albeit rare in patients, mechanistically strengthen the interaction of MCL1 with BOK, thereby increasing its antiapoptotic potential^4,10^. In addition to its antiapoptotic role, an N-terminally truncated isoform of MCL1 locates to the inner mitochondrial membrane where it facilitates adenosine triphosphate (ATP) production, preserves mitochondrial energy transduction and ensures mitochondrial homeostasis^11^.

MCL1 function and expression are tightly regulated at the transcriptional, post-transcriptional, translational and post-translational levels, rendering it a protein with a short half-life and, therefore, a need for constant replenishment^12^. Its physiologic roles in embryogenesis, cell homeostasis and lineage differentiation of hematopoietic cells have been elucidated using global and conditional MCL1 deletion models^13–16^. At the same time, MCL1 is among the most highly overexpressed pathologic proteins across all cancers^2^, including solid tumor malignancies in liver^17^, breast^18^, non-small cell lung (NSCLC)^19^, urothelial^20^ or pancreatic cancer^21^, as well as hematological cancers including acute myeloid or chronic lymphocytic leukemia^22,23^ and non-Hodgkin lymphoma^24^. The overexpression of MCL1 is commonly linked to poor prognosis^18,25,26^ and resistance to radiation therapy and chemotherapy^27–30^. Interestingly, MCL1 was demonstrated to also mediate resistance against targeted therapies including BRAF inhibitors^31^, receptor tyrosine kinase inhibitors^32,33^ and multikinase inhibitors^34,35^ that are commonly used in modern cancer therapy.

As a result of these features, MCL1 is an attractive target for cancer therapy. Inhibition or downregulation of MCL1 has been shown to restore sensitivity to various cytotoxic stimuli^36–40^. Several direct inhibitors of MCL1 function have recently been developed and are currently under investigation in early clinical trials^41–43^. However, clinical development of several programs has slowed or halted because of elevated troponin levels, which raise concerns about potential cardiotoxicity^44^. Tissue-specific *MCL1*-knockout models resulted in dysfunctional mitochondrial respiration and loss of cardiac contractility, thus demonstrating a link between MCL1 and cardiomyopathy^45^. An emerging role for MCL1 in regulating fatty acid oxidation in cardiomyocytes was also recently reported^46^. Recent in vitro studies in human induced pluripotent stem cell (hiPS cell)-derived cardiomyocytes show that prolonged exposure to various MCL1 inhibitors disrupted mitochondrial morphology and ultimately compromised cardiomyocyte functionality^44^. Taken together, these results suggest that prolonged exposure of MCL1-inhibitory agents are likely to cause on-target cardiac toxicity, calling for the need for a potent MCL1 inhibitor with sufficient exposure to induce tumor apoptosis, yet a sufficiently short half-life to spare cardiotoxicity.

Here, we introduce a new highly potent and selective MCL1 inhibitor, BRD-810, that rapidly induces apoptosis in vitro and affords strong antitumor effects in vivo. Given the short half-life of the MCL1 protein and the irreversible nature of apoptosis, we reasoned that a rapidly cleared MCL1 inhibitor would be an effective antitumor agent with potential for less toxicity than longer-acting agents. We report here our characterization of BRD-810 in tumor models and in hiPS cell-derived cardiomyocytes. The results suggest that BRD-810 is suitable for clinical development.

## Results

### BRD-810 potently binds to the BH3 groove of MCL1 protein

**Fig. 1:**
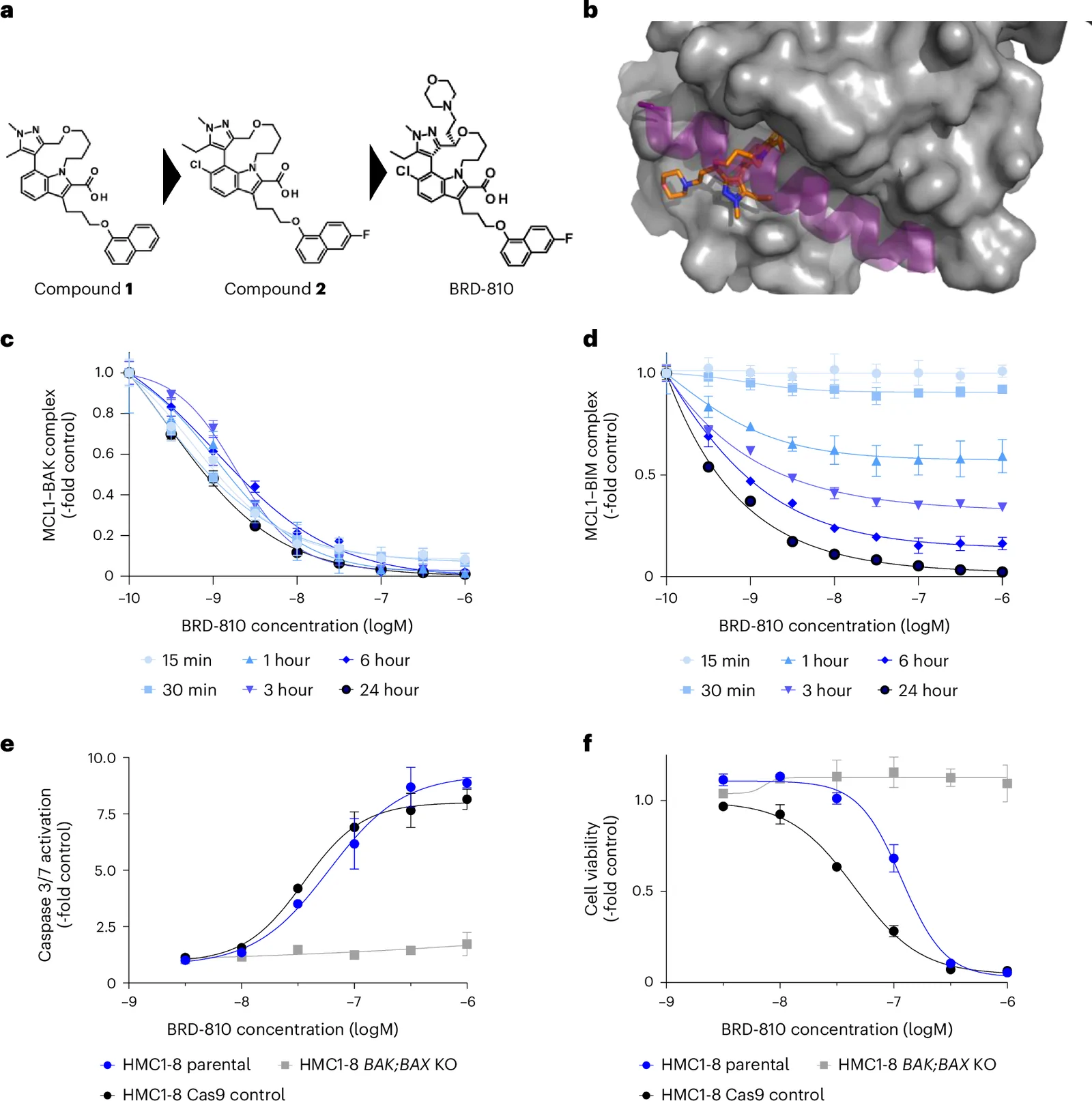
BRD-810 is a potent and selective inhibitor of the antiapoptotic protein MCL1. **a**, The key compounds from the macrocyclic MCL1 inhibitor class. **b**, Overlay of BRD-810 (orange sticks) and modified Bim BH3 peptide SAH-MS1-18 (magenta cartoon, PDB 5W89) in the BH3 binding pocket. **c**,**d**, Quantification of the MCL1–BAK (**c**) or MCL1–BIM (**d**) complex, measuring the interaction between native cellular MCL1 protein and BAK or BIM using ELISA. Data are shown as the mean and s.d. (*n* = 3 independent measurements). **e**,**f**, Activation of caspases 3 and 7 (**e**) and cell viability (**f**) under increasing doses of BRD-810 in parental HMC1-8 cells, CRISPR-edited *BAK*;*BAX*-knockout cells and HMC1-8-Cas9-expressing control cells. Data are shown as the mean and s.d. (*n* = 2 independent measurements). Source data

BRD-810 was identified during a medicinal chemistry campaign tasked with balancing cellular potency and in vivo clearance within a macrocyclic MCL1 inhibitor class. The three key compounds from this optimization effort are summarized in Fig. 1a and Table 1. The compound class was identified to bind reversibly to human MCL1 protein and block the interaction between MCL1 and a peptide derived from the BH3-only protein Noxa in a homogeneous time-resolved fluorescence (HTRF) assay (half-maximal inhibitory concentration (IC_50_) = 1.7 nM for compound **1**). In addition, the compound class showed selectivity for MCL1 over other antiapoptotic proteins such as Bcl-X_L_ and Bcl-2, as none of the key compounds affected the interaction of those other Bcl-2 family members with their respective BH3-only protein in biochemical HTRF assays (up to 20 µM). BRD-810 showed no appreciable off-target binding versus a broad panel of enzymes, receptors and ion channels (PanLabs SafetyScreen panel, provided as Supplementary Table 1; Eurofins Discovery). Lead optimization starting from compound **1** achieved a 20-fold improvement in MCL1 protein-binding affinity by introducing halogen substituents to both the indole and the naphthalene moieties (compound **2**, dissociation constant *K*_d_ = 1.2 nM). The cellular potency of compound **2** was notably enhanced by the introduction of a morpholino-ethyl side chain adjacent to the oxygen atom of the macrocycle, yielding a compound we named BRD-810 (unbound IC_50_ (IC_50u_) = 0.3 nM in AMO-1 cells).

**Table 1:** Overview of compound optimization route toward the identification of BRD-810

	Compound 1	Compound 2	BRD-810
SPR (nM, *K*_d_)	21.4	1.2	0.3
MCL1–Noxa HTRF (nM, IC_50_)	1.7	0.8	0.4
Bcl-X_L_–Bad HTRF (nM, IC_50_)	>20,000	>20,000	>20,000
Bcl-2–Bad HTRF (nM, IC_50_)	>20,000	>20,000	>20,000
Caspase 3 activation (EC_50_, nM)	2,500	90	7
AMO-1 antiproliferative IC_50u_ (nM)	1,100	3	0.3
HMC1-8 antiproliferative IC_50u_ (nM)	>7,000	11	2.3
In vivo rat intravenous MRT (h)	1.4	0.6	0.3

The table presents the structure–activity relationship characterization data reflecting the improvement of BRD-810 toward high potency and rapid clearance. Data are given as the rounded mean of *n* = 3 independent experiments.

**Extended Data Fig. 1:**
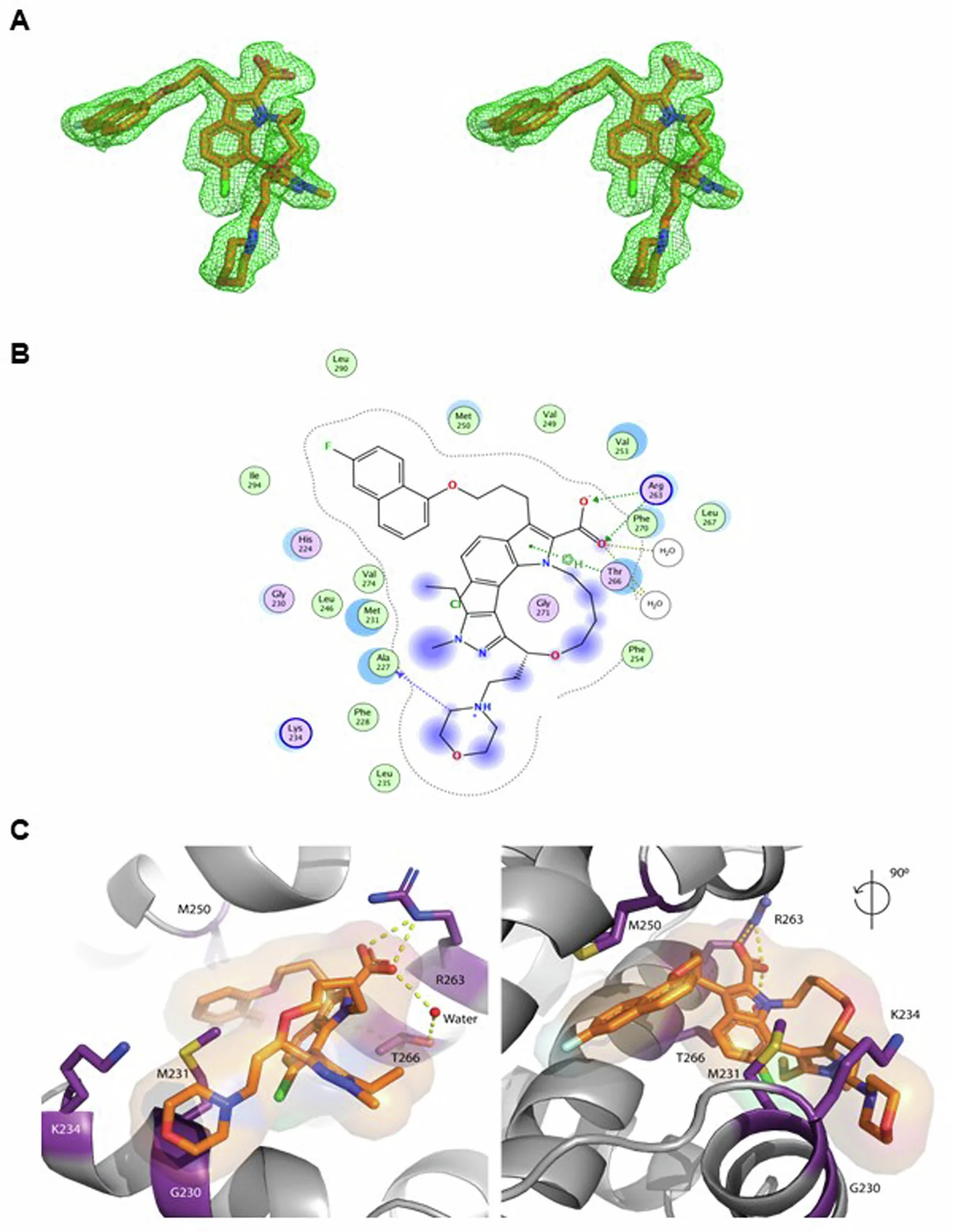
Crystal structure of BRD-810 binding MCL1. **A**, Electron density for BRD-810 bound to MBP-MCL1. Stereo view of Fo-Fc Omit map for BRD-810 contoured at 2.0σ. **B**, 2D ligand interactions map of BRD-810 bound to MCL1. 2D Ligand interactions are generated using MOE software. **C**, Active site ligand interactions. Left panel: Front view of ligand interactions in binding pocket. Right panel: Side view (90^o^) of ligand interactions in binding pocket. BRD-810 is represented in orange sticks, MCL1 residues interacting with the ligand in purple sticks and the water bridging molecule in red small sphere. Figure generated with Pymol.

Our medicinal chemistry campaign benefitted from a previously described crystallography platform^47^ that generated experimentally confirmed binding poses of >50 unique compounds to guide iterative design and testing. BRD-810 occupies the BH3 binding groove of MCL1 (Fig. 1b and Supplementary Table 2), as evidenced by the unambiguous electron density and a lack of interactions with symmetry-related protein molecules (Extended Data Fig. 1a–c). The naphthalene ring embeds deeply in an induced binding pocket largely created by the movement of M250, with the fluorine atom lying at the bottom of this pocket. The BRD-810 carboxylate engages R263 with bidentate hydrogen bonds, as well as a water-mediated hydrogen-bond bridge to T266. The additional morpholino group of BRD-810 packs against helix 3, predominantly involving G230, M231 and K234.

In addition to a tenfold greater cellular potency compared to compound **2**, BRD-810 was more rapidly cleared following intravenous administration to rats (mean residence time (MRT) = 0.3 h versus MRT = 0.6 h for compound **2**). Collectively, these findings suggest BRD-810 can potently and selectively block proapoptotic proteins from binding MCL1 before being rapidly cleared from the system.

### MCL1 inhibitor BRD-810 triggers apoptosis in cancer cells

**Extended Data Fig. 2:**
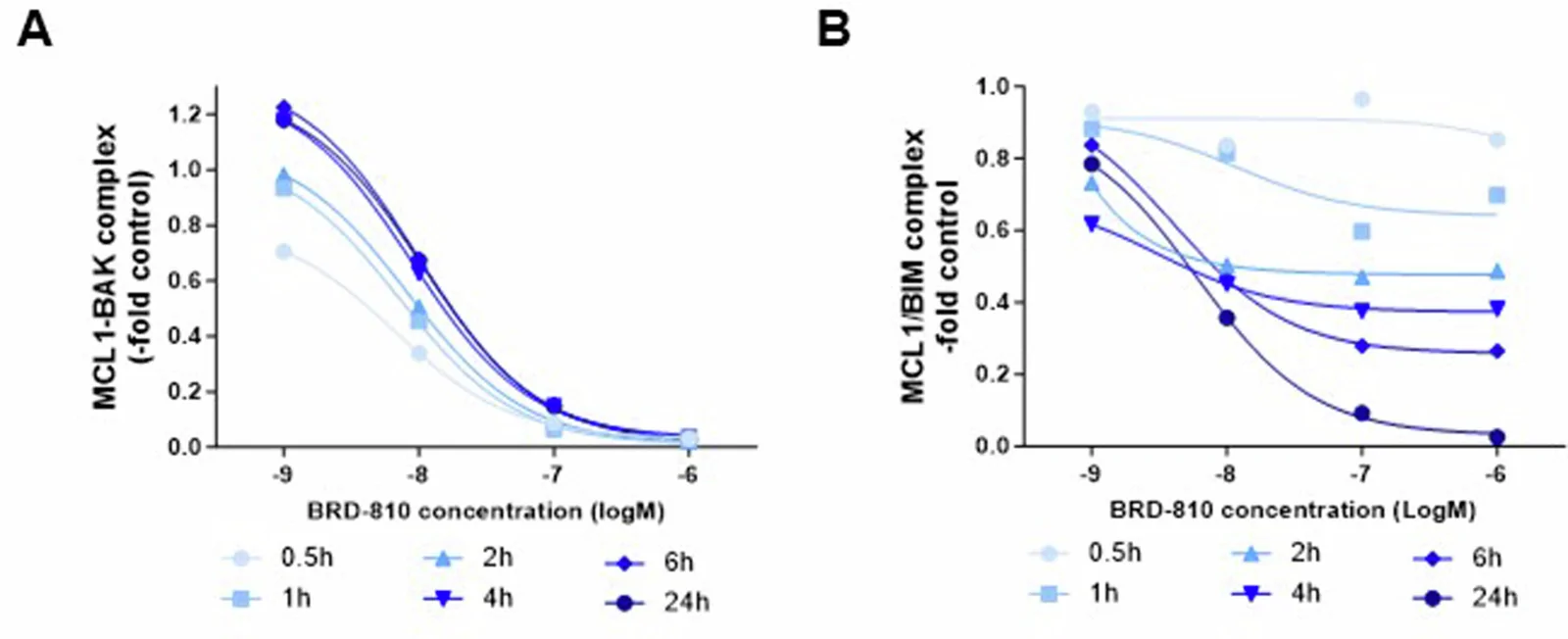
Co-immunoprecipitation of MCL1-BAK and MCL-Bim. MCL-1 protein was co-immunoprecitated from cells treated with increasing concentrations of BRD-810 for the time indicated. Immunoblots were stained for the respective binding partner BAK (**A**) and BIM (**B**) and bands were quantified using densitometric analysis. Data is shown as single measurement per timepoint and concentration.

To investigate the potential of BRD-810 to displace the complexes of MCL1 and proapoptotic proteins in intact cells, we developed a quantitative ELISA assay. This assay measures the interaction of native MCL1 protein and BAK or BIM protein at increasing doses of BRD-810 and at different exposure time points. Short-term treatment (as early as 15 min) with BRD-810 blocked the interaction of MCL1 with BAK in a dose-dependent manner (Fig. 1c) and the IC_50_ of BRD-810 required to disrupt the MCL1–BAK complex in cells was calculated to be 1.2 nM on average. In contrast, disruption of the MCL1–BIM complex by BRD-810 was clearly dose and time dependent (Fig. 1d) and longer exposure times were required to fully disrupt the complex, reflecting the longer target residence time of BIM on MCL1 protein^48^. Co-immunoprecipitation assays showed similar complex disruption dynamics when cells were exposed to increasing concentrations of BRD-810 (Extended Data Fig. 2a,b).

We next investigated the downstream cellular effects of disruption of MCL1 and proapoptotic protein complexes. Treating the breast cancer cell line HMC1-8 with increasing concentrations of BRD-810 led to the activation of caspase 3 and induction of cell death. These effects were dose dependent and occurred at comparable concentrations of BRD-810 (half-maximal effective concentration (EC_50_) = 66 nM for caspase activation and IC_50_ = 63 nM for cell growth inhibition; Fig. 1e,f). To determine whether BRD-810 induced cell killing through the intrinsic apoptotic pathway, we generated BAX-deficient and BAK-deficient HMC1-8 cells using clustered regularly interspaced short palindromic repeats (CRISPR)–Cas9 gene editing to compare those double-knockout cells to their isogenic HMC1-8 Cas9 control cells with regard to their sensitivity to BRD-810. While the HMC1-8 Cas9 control cells behaved like the parental HMC1-8 cells, knockout of *BAX* and *BAK* protected cells from BRD-810-mediated induction of apoptosis and cell killing. This suggests that BRD-810-induced cell death indeed occurs through an on-target mechanism activating the intrinsic apoptotic pathway (Fig. 1e,f).

To demonstrate the MCL1 specificity of BRD-810 in intact cells, we measured caspase activation and growth inhibition in diffuse large B cell lymphoma (DLBCL) lines known to be either MCL1 or Bcl-X_L_ dependent. Treatment of SU-DHL-5 and SU-DHL-10 cells (MCL1-dependent cell lines) with increasing doses of BRD-810 led to potent (caspase EC_50_ activation and IC_50_ cell growth inhibition < 10 nM) and rapid (within ~4 h) induction of apoptosis, while in Bcl-X_L_-dependent lines such as SU-DHL-4 cells, apoptosis was only observed at >600-fold higher concentrations (Supplementary Table 3). In sum, these findings establish BRD-810 as a selective MCL1 inhibitor.

### Candidate biomarker for BRD-810 susceptibility

**Fig. 2:**
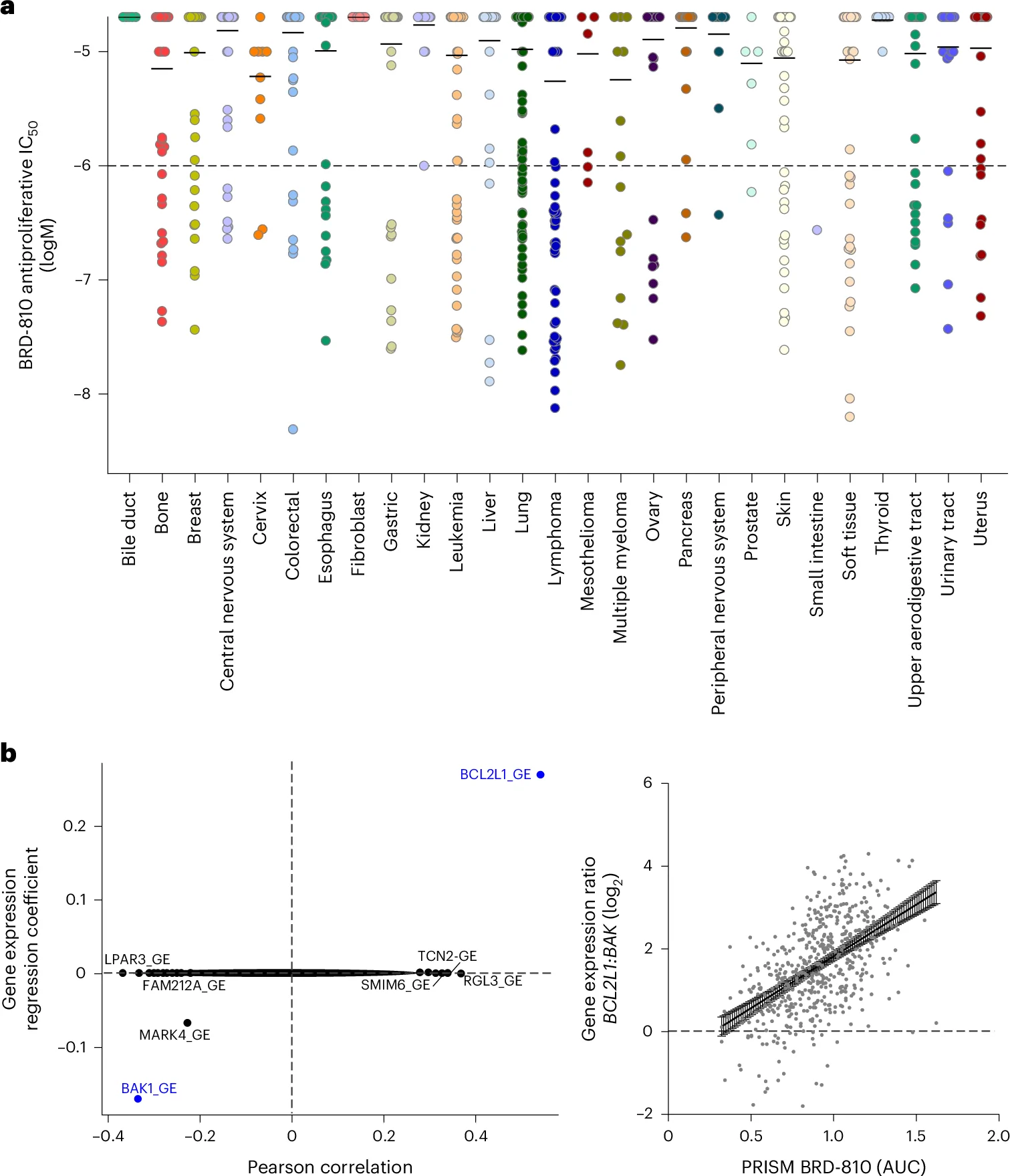
BRD-810 has anticancer efficacy over a broad range of cancer types in vitro. **a**, Antiproliferative IC_50_ values from dose–response curves generated by exposing over *n* = 700 barcoded cancer cell lines ordered by cancer type to increasing doses of BRD-810 for 5 days (PRISM screen; each dot is the BRD-810 IC_50_ for one cell line and represents one measurement; black lines indicate the median per cancer type). **b**, Unbiased, genome-wide gene expression correlative to drug activity determined using Bayesian variable selection methods; the posterior mean regression coefficients are plotted against the Pearson correlation between each feature and the AUC of 700 cell lines (*n* = 700 cell lines; error bars represent the 95% confidence interval). Source data

**Extended Data Fig. 3:**
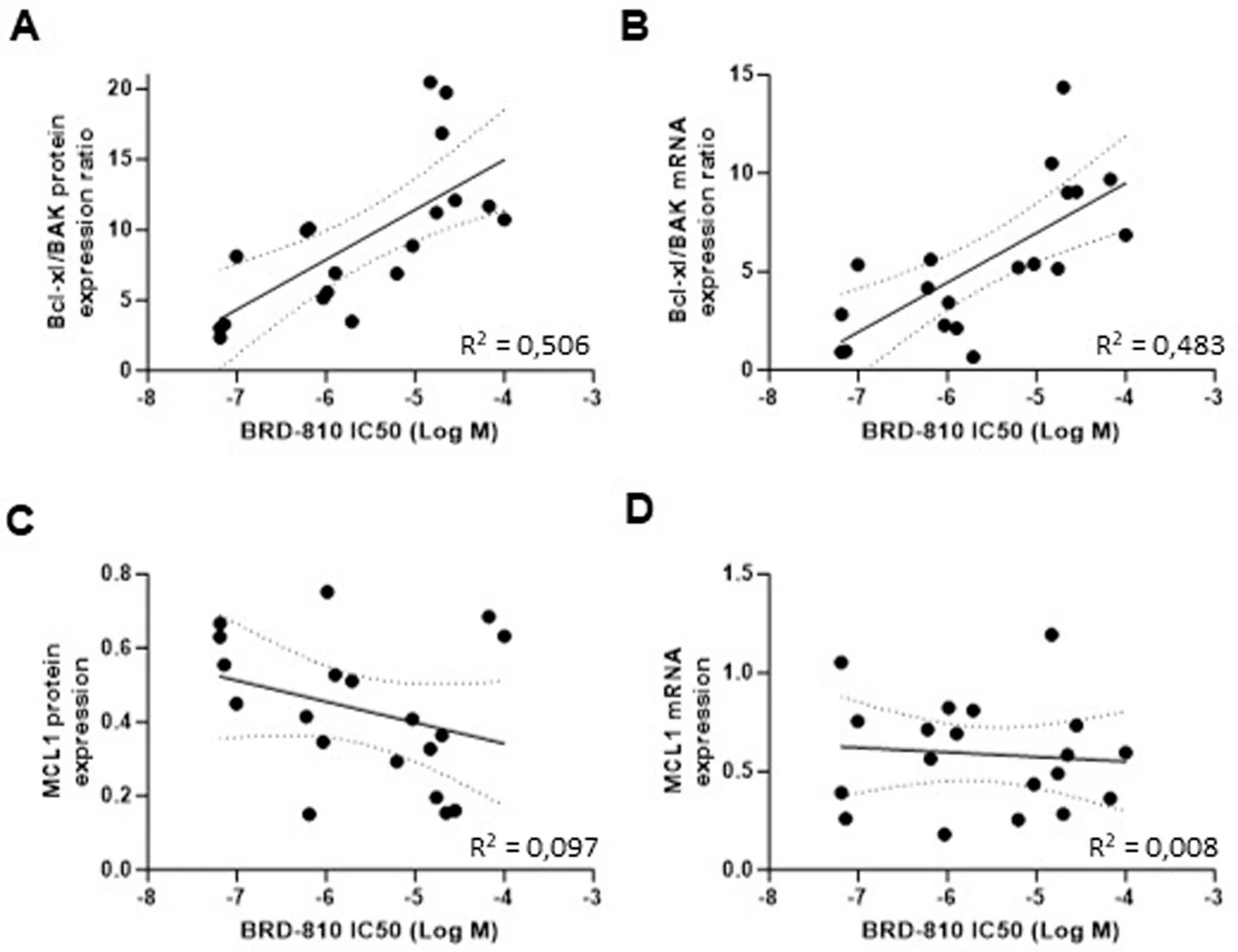
Correlation of Bcl-xl, BAK and MCL1 protein or mRNA expression to BRD-810 sensitivity in breast cancer cell lines. The expression level ratio of Bclxl and BAK protein (**A**) or mRNA (**B**), or MCL1 protein (**C**) or mRNA (**D**) was correlated to the sensitivity of breast cancer cell lines. Data is shown as linear regression with 95% CI (n = 19 cell lines). Source data

To comprehensively evaluate the antiproliferative activity of BRD-810, we used a rapid, high-throughput multiplex screen called PRISM^49^. The PRISM method enables pooled screening of over 700 barcoded cell lines representing 32 different cancer lineages (Supplementary Table 4)^50^. Following a 5-day incubation of cell line pools with BRD-810, antiproliferative activity was observed at submicromolar concentrations across a broad range of solid and hematological cancer models, including breast cancer, lung cancer, melanoma, sarcoma, lymphoma and leukemia (Fig. 2a). To better understand the biological drivers of BRD-810 specificity, we used Bayesian variable selection methods to identify a subset of baseline genomic features of cell lines that best explained the measured MCL1 inhibitor sensitivity and found that low levels of *BCL2L1*, coding for Bcl-X_L_, and high levels of *BAK* expression correlated most strongly with BRD-810-induced efficacy (Fig. 2b), with the ratio of *BCL2L1* to *BAK* mRNA levels providing the best correlation across all cell lines (Fig. 2b). Further validating this finding in a panel of breast cancer cell lines, we confirmed that BRD-810 sensitivity strongly correlated with the *BCL2L1*:*BAK* expression ratio using mRNA or protein quantification (Extended Data Fig. 3a,b). Interestingly, sensitivity was less correlated with MCL1 protein or mRNA expression (Extended Data Fig. 3c,d), suggesting that MCL1 dependency is more predicted by how much Bcl-X_L_ is present to compensate for blocked MCL1 protein, as well as how much proapoptotic BAK, the preferred binding partner to MCL1 and Bcl-X_L_, can be freed by MCL1–BAK disruption.

We also compared BRD-810 to other MCL1 inhibitors that have entered clinical trials. In MCL1 and other Bcl-2 family inhibitor classes, overcoming nonspecific binding to plasma proteins is a major design challenge and a wide range of protein binding is observed between structurally distinct BH3 mimetics. To enable intrinsic potency comparisons, the unbound fraction under cell culture conditions was measured for all MCL1 inhibitors, as only the unbound fraction of each compound is presumed to exert a biologic effect (Table 2). We selected cell lines identified to be BRD-810 sensitive in the PRISM screen across a range of hematological and solid tumor cancer types and treated them with MCL1 inhibitors BRD-810, AZD5991, AMG176 or S64315 to evaluate their antiproliferative IC_50_ values in a head-to-head comparison. We found that all cell lines from the PRISM screen were indeed MCL1 inhibitor sensitive and BRD-810, when corrected for the unbound fraction, was the most potent MCL1 inhibitor among those tested (Table 2 and Supplementary Table 5).

**Table 2:** Antiproliferative activity of BRD-810 and other MCL1 inhibitors

	BRD-810	AMG176	AZD5991	S64315
Cell culture medium unbound fraction (%)	3.1	1.0	2.1	52.2
Antiproliferative IC_50u_ (nM)				
*Hematological cancer cell lines*				
SU-DHL-10	0.1	0.9	ND	1.6
SU-DHL-5	0.2	1.2	0.3	0.5
KMS-12-BM	0.3	1.9	0.7	ND
AMO-1	0.3	1.3	1.2	1.7
RPMI 8226	0.3	3.0	1.3	ND
OPM-2	0.4	1.2	0.7	2.7
MOLP-8	0.5	2.3	0.8	3.9
JJN-3	0.9	5.7	1.5	3.5
KMS-12-PE	1.5	12.0	4.4	ND
*Solid cancer cell lines*				
DMS114	0.6	2.9	1.5	2.9
A-427	0.6	5.8	1.1	7.3
HCC-1187	0.9	3.4	1.1	2.6
SNU398	1.1	5.1	1.1	3.7
HCC-2157	1.4	2.7	2.0	4.2
PA-1	1.8	6.5	2.2	9.2
NCI-H82	1.8	4.9	3.4	19.1
SNU-16	2.0	6.0	5.6	38.0
A-431	2.2	6.6	4.6	37.0
HMC1-8	2.3	6.0	6.9	23.0

The table presents the free fraction of BRD-810 and other MCL1 inhibitors that have entered clinical trials (AMG176, AZD5991 and S64315) in cell culture medium with 10% fetal calf serum, determined using a protein-binding assay with equilibrium dialysis. Cell viability assays were performed for each cell line to confirm PRISM screen data. Cell lines were exposed to increasing doses of BRD-810 or other MCL1 inhibitors as tumor indications and the antiproliferative IC_50_ after 72 h was determined. IC_50_ values were corrected by the free fraction to gain the unbound IC_50_ (IC_50u_) values shown in the table. Data are given as the rounded mean (*n* = 2 independent measurements).

### Cell killing dynamics of BRD-810

**Fig. 3:**
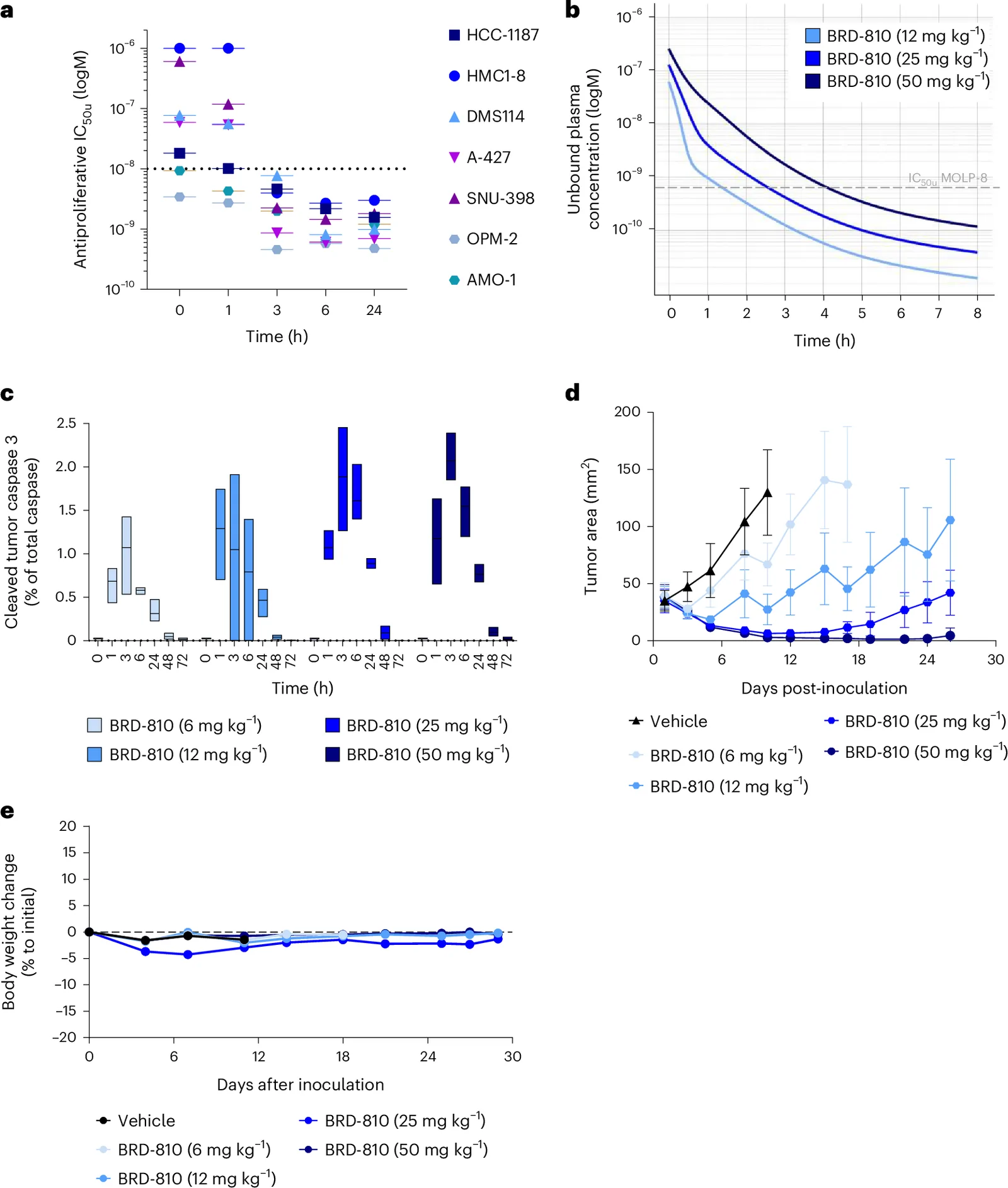
Dynamics of BRD-810-induced killing suggests a short-term 1-h coverage of unbound IC_50_ sufficient to induce antitumor efficacy in vivo. **a**, Cancer cells were exposed to increasing concentrations of BRD-810 for 5 min, 1 h, 3 h or 6 h before the compound was washed off and replaced by medium or cancer cells were continuously exposed for 24 h. Antiproliferative IC_50_ values were determined at 24 h and corrected for the free fraction of BRD-810 in the cell culture medium to get the IC_50u_. Data are shown as individual measurements of independently performed experiments (*n* = 7 cell lines per time point). **b**, BRD-810 plasma concentrations in blood from immunocompromised mice that were intravenously injected with BRD-810. The solid line indicates the modeled exposure data based on a mouse-PK model; the dashed line indicates the in vitro antiproliferative IC_50u_ of BRD-810 in MOLP-8 cells. **c**, BRD-810 was intravenously injected into MOLP-8 tumor-xenografted mice. Tumors were sampled at the indicated time points and the total caspase 3 and cleaved caspase 3 levels were measured by western blot using human-specific antibodies and subsequently quantified by densiometric analysis. Data are shown as the median and quartiles (*n* = 3 tumors per dose and time point). **d**,**e**, Antitumor efficacy (**d**) and body weight (**e**) of MOLP-8 tumor-xenografted mice (*n* = 10 mice per group) after once-weekly intravenous injections of BRD-810. Data are shown as the mean and s.d. Source data

Having established the spectrum and specificity of BRD-810 cell killing, we next embarked on experiments to understand the time and dose dependency of BRD-810-induced cell killing. This is important because the ideal MCL1 inhibitor would provide sufficient exposure to induce MCL1-dependent tumor cell death but not beyond that so as to minimize on-target toxicity. MCL1-dependent cell lines were treated with varying concentrations of BRD-810 for 5 min (indicated as 0 h), 1 h, 3 h, 6 h or 24 h and then thoroughly washed to remove compound from cells. Antiproliferative IC_50_ values were determined at the 24-h time point (Fig. 3a). At exposure durations of 1 h or less, the unbound antiproliferative IC_50_ for most cell lines was well above 10 nM, indicating that short-term exposures would require a large amount of BRD-810 to induce efficient antiproliferative effects. However, by 3 h of exposure, cancer cell death was profound, with IC_50u_ values below 10 nM for all MCL1-dependent cell lines. Importantly, exposure times longer than 6 h and up to 24 h resulted in no added benefit. This result indicates that ~4 h of exposure is sufficient for MCL1-mediated tumor cell killing and further exposure is likely to contribute primarily to toxicity not efficacy.

### In vivo efficacy of BRD-810

From the rapid cell-killing dynamics identified in vitro, we hypothesized that a high-clearance MCL1 inhibitor would be able to initiate apoptosis and exert in vivo efficacy. Pharmacokinetic characterization of BRD-810 revealed its rapid clearance with a half-life of 0.6 h in mice (Fig. 3b), which translates into a predicted half-life in human of 1.1 h using allometric species scaling. To characterize the proximal pharmacodynamic effects of BRD-810 in vivo, mice engrafted with MOLP-8 multiple myeloma cells were injected with a single dose of 6, 12.5, 25 or 50 mg kg^−1^ of BRD-810 and then subjected to tumor biopsy at 1, 3, 6, 24, 48 and 72 h to allow the measurement of cleaved caspase 3 levels as an indicator of apoptosis (Fig. 3c). Cleaved caspase 3 levels could be detected at all doses as early as 1 h and up to 6 h after BRD-810 intravenous injection, indicating a rapid induction of apoptosis that was sustained even after the compound was cleared from the plasma by the hepatobiliary transport systems. The maximum caspase induction occurred approximately 3 h following BRD-810 injection and this time dependency was similar across all doses tested. However, the magnitude of caspase 3 activation increased up to 25 mg kg^−1^ in a dose-dependent manner and was similar from 25 to 50 mg kg^−1^ suggesting that a maximum functional effect was achieved.

Having established that the rapidly cleared BRD-810 is sufficient to maximally induce apoptosis in vivo, we next evaluated the antitumor efficacy of BRD-810 in the same MOLP-8 tumor model (Fig. 3d). Once-weekly dosing of BRD-810 led to a dose-dependent inhibition of tumor growth, again with a maximum effect reached at 25 mg kg^−1^. Like most MCL1 inhibitors, BRD-810 does not inhibit murine Mcl1; as such, the on-target toxicity of BRD-810 cannot be assessed in mice. However, as one measure of off-target toxicity, we observed no impact on body weight following once-weekly dosing (Fig. 3e).

**Fig. 4:**
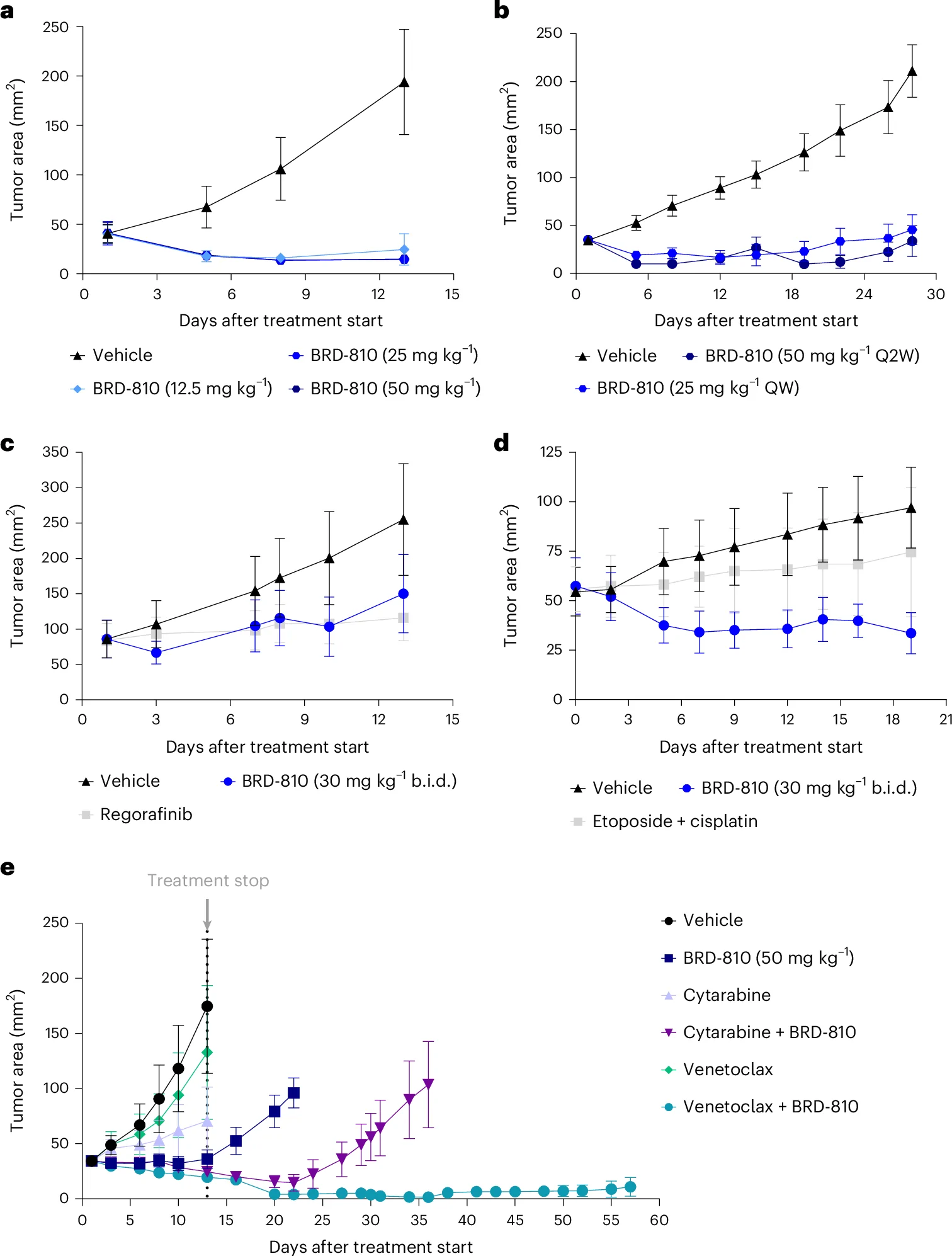
In vivo efficacy of BRD-810 in hematologic and solid tumor xenograft models. **a**, BRD-810 was administered as a single intravenous injection to AMO-1 (multiple myeloma) tumor-bearing mice (*n* = 10 mice per group). **b**, BRD-810 was intravenously injected once weekly at 25 mg kg^−1^ and once every second week at 50 mg kg^−1^ to SU-DHL-10 (DLBCL) xenografted mice (*n* = 12 mice per group). **c**,**d**, BRD-810 was given as two intravenous injections 2 h apart to mice bearing SNU398 (HCC) xenografts (**c**; *n* = 7 mice per group) or A-427 (NSCLC) xenografts (**d**; *n* = 7 mice per group). Regorafinib was given orally at 30 mg kg^−1^ once daily. Etoposide was administered at 12 mg kg^−1^ (3 days on, 11 days off) and in combination with 6 mg kg^−1^ cisplatin once weekly as intraperitoneal injections. **e**, THP-1 (AML) xenografted mice (*n* = 10 mice per group) were treated with once-weekly intravenous injections of BRD-810 in monotherapy or in combination with 100 mg kg^−1^ venetoclax given orally, daily or in combination with 45 mg kg^−1^ cytarabine given as a subcutaneous injection (3 days on, 4 days off). All data are shown as the mean and s.d. Source data

To understand the broader, single-agent potential of BRD-810 across different cancers, we investigated the efficacy of BRD-810 in two hematological and two solid tumor xenograft models. In the rapidly growing multiple myeloma xenograft model AMO-1, a single dose of BRD-810 as low as 12.5 mg kg^−1^ induced complete response within 12 days (Fig. 4a). In the SU-DHL-10 DLBCL xenograft model, we compared once-weekly dosing of 25 mg kg^−1^ to 50 mg kg^−1^ given every second week and found that less frequent, higher doses of BRD-810 led to prolonged tumor regression in this model (Fig. 4b).

For solid tumor xenograft models, the SNU398 (liver) and A-427 (NSCLC) tumor models were chosen as representative solid tumor malignancies. Xenografted mice were treated with two successive bolus injections of 30 mg kg^−1^ (2 h apart) to achieve the desired 4-h coverage of the unbound IC_50_ in each cell model. Using a schedule of 2 days on and 5 days off, BRD-810 showed comparable efficacy to standard of care (regorafenib) in the SNU398 liver cancer model (Fig. 4c). Using the same treatment regimen for the NSCLC model A-427, we found that BRD-810 triggered strong tumor regression to a greater extent than the combination of the standard chemotherapy agents cisplatin and etoposide (Fig. 4d).

**Extended Data Fig. 4:**
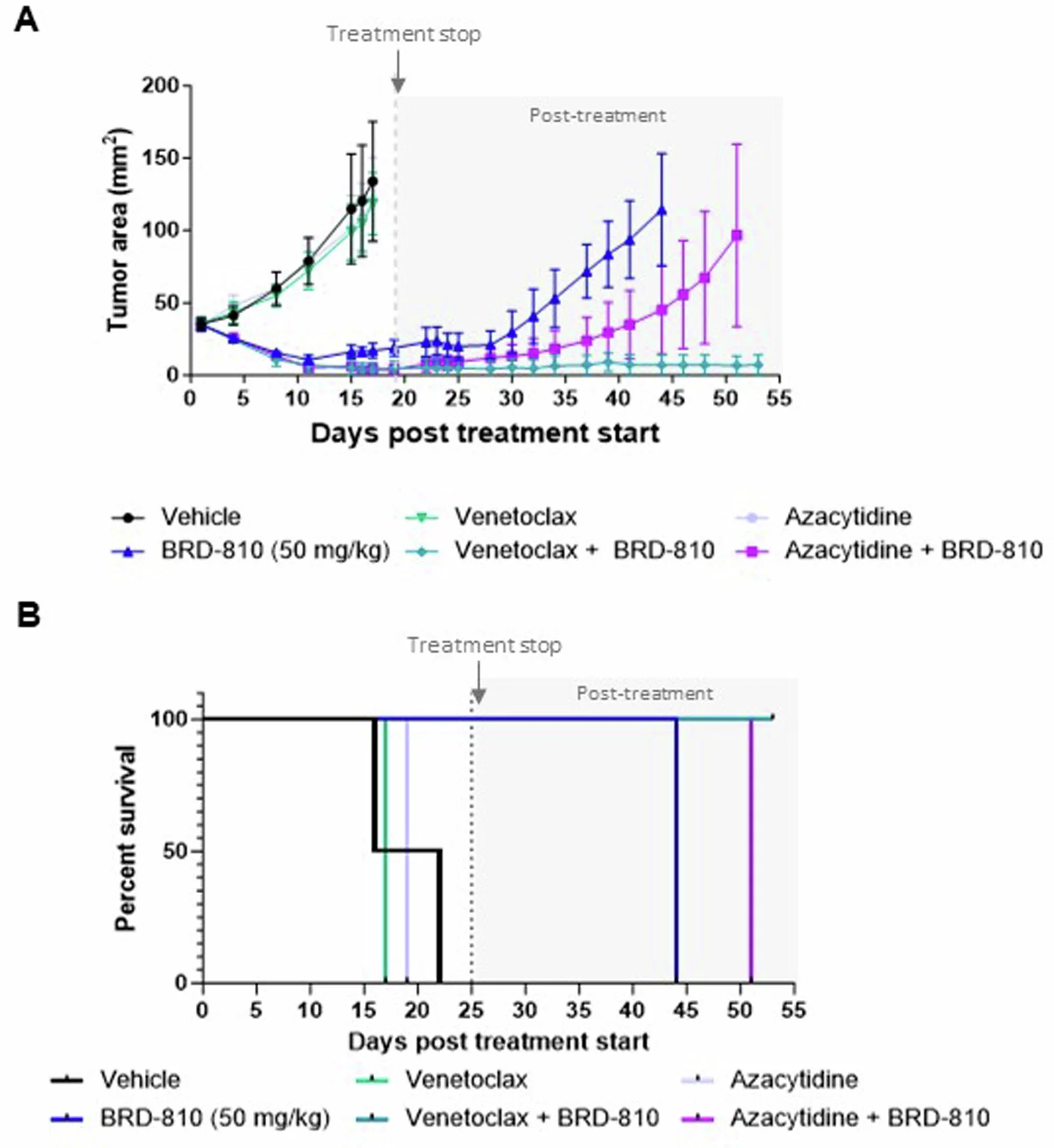
In vivo efficacy and overall survival in AML xenografted mice. **A**, MV-4-11 (AML)-xenografted mice (n = 10 mice per group) were treated with once weekly intravenous injections of 50 mg/kg BRD-810 in monotherapy or in combination with 100 mg/kg venetoclax given orally, daily or in combination with Azacytidine 3 mg/kg given as intraperitoneal injections 5on2off. All data is shown as mean and SD. **B**, Survival was tracked for all groups (n = 10 mice per group) over time even after stop of treatment. Source data

Given the role of MCL1 in promoting resistance to conventional and targeted therapy, we also investigated the antitumor effects of BRD-810 in treatment-refractory settings, both as a single agent and in combination with standard of care. In the THP-1 model of acute myeloid leukemia (AML), neither the Bcl-2 inhibitor venetoclax nor cytarabine showed appreciable single-agent efficacy (Fig. 4e) closely resembling the clinical experience in refractory AML patients^51^. In contrast, weekly dosing of 50 mg kg^−1^ BRD-810 had robust antitumor effects in monotherapy. When given in combination with cytarabine, the efficacy of BRD-810 improved, with no tumor outgrowth observed for 2 weeks after treatment. While others have previously established that venetoclax monotherapy induces MCL1 levels and confers resistance^51^, the combination of BRD-810 and venetoclax was synergistic and able to elicit complete response in all animals, with no tumor recurrence during the 4 weeks of post-treatment monitoring. Similar efficacy was seen in MV-4-11 xenografted mice, in which BRD-810 combination with either azacytidine or venetoclax led to notably improved overall survival even after the treatment was stopped (Extended Data Fig. 4a,b)

### Effect of BRD-810 on hiPS cell-derived cardiomyocytes

**Fig. 5:**
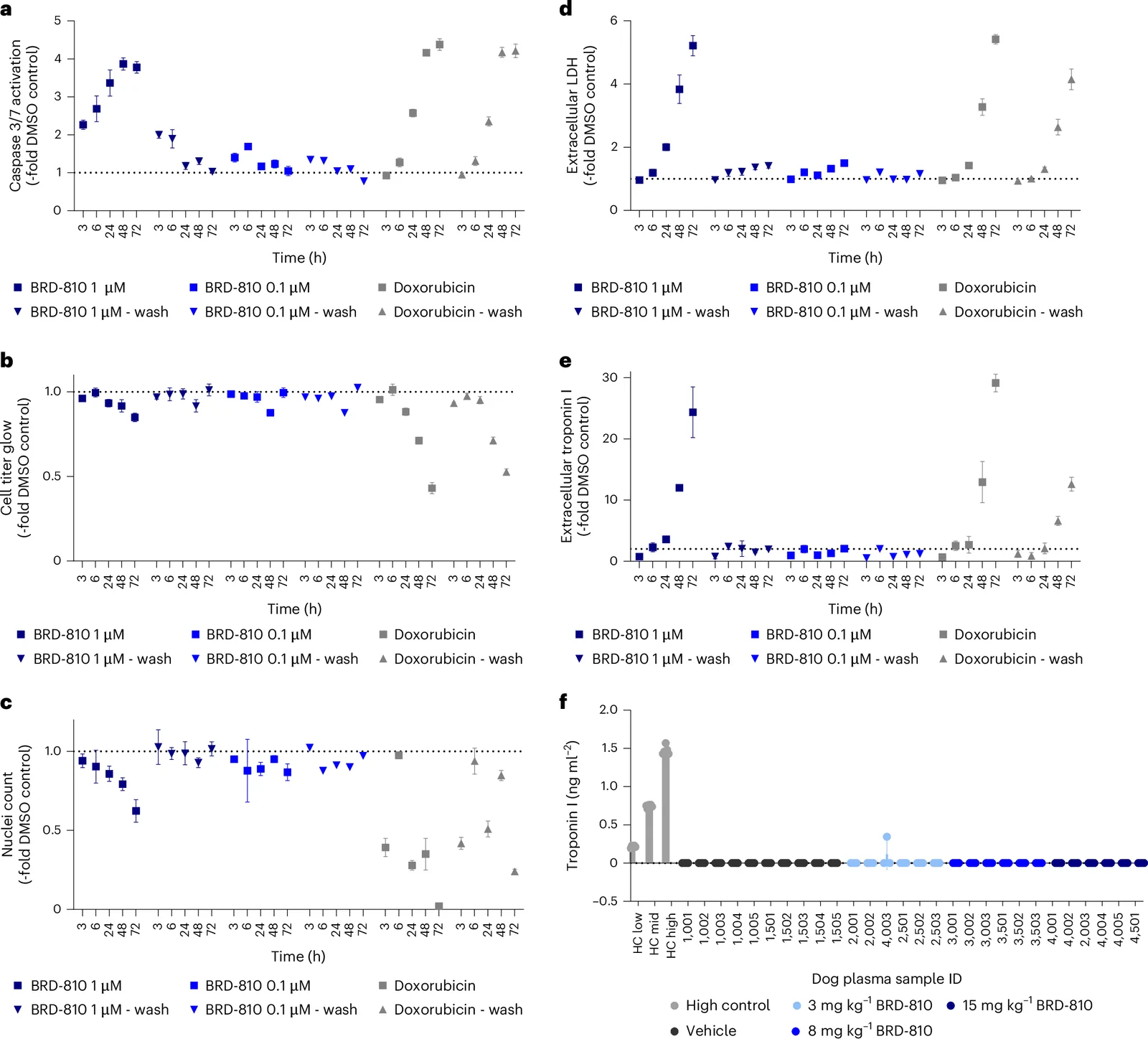
Effect of BRD-810 on iPS cell-derived cardiomyocytes. **a**–**e**, iPS cell-derived cardiomyocytes were exposed to BRD-810 or doxorubicin continuously for 72 h or 4 h, after which compounds were washed off (‘wash)’ and the activation of caspases 3 and 7 (**a**), cell viability according to cell titer glow (**b**), DAPI-stained number of nuclei (**c**), extracellular LDH (**d**) or extracellular troponin I (**e**) was determined at the indicated time points. All data are shown as the mean and s.d. (*n* = 3 independent measurements). **f**, Beagle dogs (*n* = 6 beagle dogs per group; three female and three male) were intravenously infused with BRD-810 for 4 h once weekly for 4 weeks and troponin I levels were determined in the blood 24 h after the final dose was given. Data are shown as triplicate measurements per dog. Source data

The effects of MCL1 inhibitors on normal cardiomyocytes are important to document in light of early clinical experience with MCL1 inhibitors^44^. An essential role for MCL1 in cardiomyocyte function has recently been proposed and in vitro studies using BH3 mimetics showed impaired survival of hiPS cell-derived cardiomyocytes following prolonged exposure^52,53^. Therefore, we assessed the effect of BRD-810 on hiPS cell-derived cardiomyocytes with a focus on the anticipated duration of exposure of BRD-810 in humans. As a positive control for these hiPS cell studies, we treated cells with doxorubicin, a chemotherapeutic agent known to induce cardiotoxicity in humans^54^. hiPS cell-derived cardiomyocytes were treated with 1 µM doxorubicin or two dose levels of BRD-810 for 72 h. Caspase activation was measured as an indicator of apoptosis, as were three markers of cell viability (ATP levels, lactate dehydrogenase (LDH) release and nuclear counting) and a tissue-specific marker of cardiac injury (troponin I) (Fig. 5a–e). As expected, doxorubicin induced a time-dependent activation of caspase 3 and reduced the number of viable hiPS cell-derived cardiomyocytes, as indicated by increased LDH release, reduced ATP levels and decreased nuclear counts (Fig. 5a–d). Prolonged exposure of hiPS cell-derived cardiomyocytes to suprapharmacological levels of BRD-810 (1 µM; tenfold higher than efficacious concentrations) led to activation of caspase 3 and increased levels of released LDH at levels comparable to doxorubicin (Fig. 5a,d). In contrast, no change from baseline was detected for ATP levels, LDH release or nuclear counts following prolonged exposure of BRD-810 at lower, yet still efficacious doses (0.1 µM), indicating the potential for a viable therapeutic margin in the MCL1 target class^41^.

We next examined the impact of shorter exposure times of doxorubicin and BRD-810 to hiPS cell-derived cardiomyocytes. Limiting the exposure of iPS cell-derived cardiomyocytes to doxorubicin to 4 h led to similar effects to the continuous exposure. In contrast, 4-h exposure to 0.1 µM or even 1 µM BRD-810 resulted in no cardiomyocyte toxicity according to any of the five measures of cardiac toxicity (Fig. 5a–e). This result suggests that the therapeutic window for MCL1 inhibitors such as BRD-810 can be further expanded by limiting the exposure time to the minimum time required for efficacy and that compounds with fast clearance profiles may best allow for such transient inhibition. To further explore the cardiac safety profile of BRD-810, we performed short-term infusion studies in dogs. Dogs are a particularly relevant preclinical species to characterize the potential on-target toxicity of MCL1 inhibitors such as BRD-810 because, in contrast to rodent Mcl1, canine MCL1 is highly similar to human MCL1 (refs. ^55,56^). Dogs were infused over a 4-h period to generate plasma levels of BRD-810 that were sufficient for in vivo efficacy in multiple murine tumor models, with 3, 8 and 15 mg kg^−1^, corresponding to in vitro cell culture concentrations of 0.5, 1 and 2 µM, respectively. This 4-h infusion was repeated once weekly for a total of 4 weeks and troponin I levels were measured at the end of the study. No troponin I was detected in the blood of any of the dogs at mid or high dose. In the low-dose group, a slight elevation of troponin I was observed in one of three replicate measurements in dog 4003 (0.000, 0.344 and 0.000 ng ml^−1^). The same animal was noted during pretreatment electrocardiogram (ECG) recordings to have a QRS fragmentation^53^. Overall, the lack of troponin I increase with short-term in vivo infusion of BRD-810 augments in vitro findings from hiPS cell-derived cardiomyocytes that short-term exposure to BRD-810 is a viable therapeutic approach to MCL1 inhibition.

**Fig. 6:**
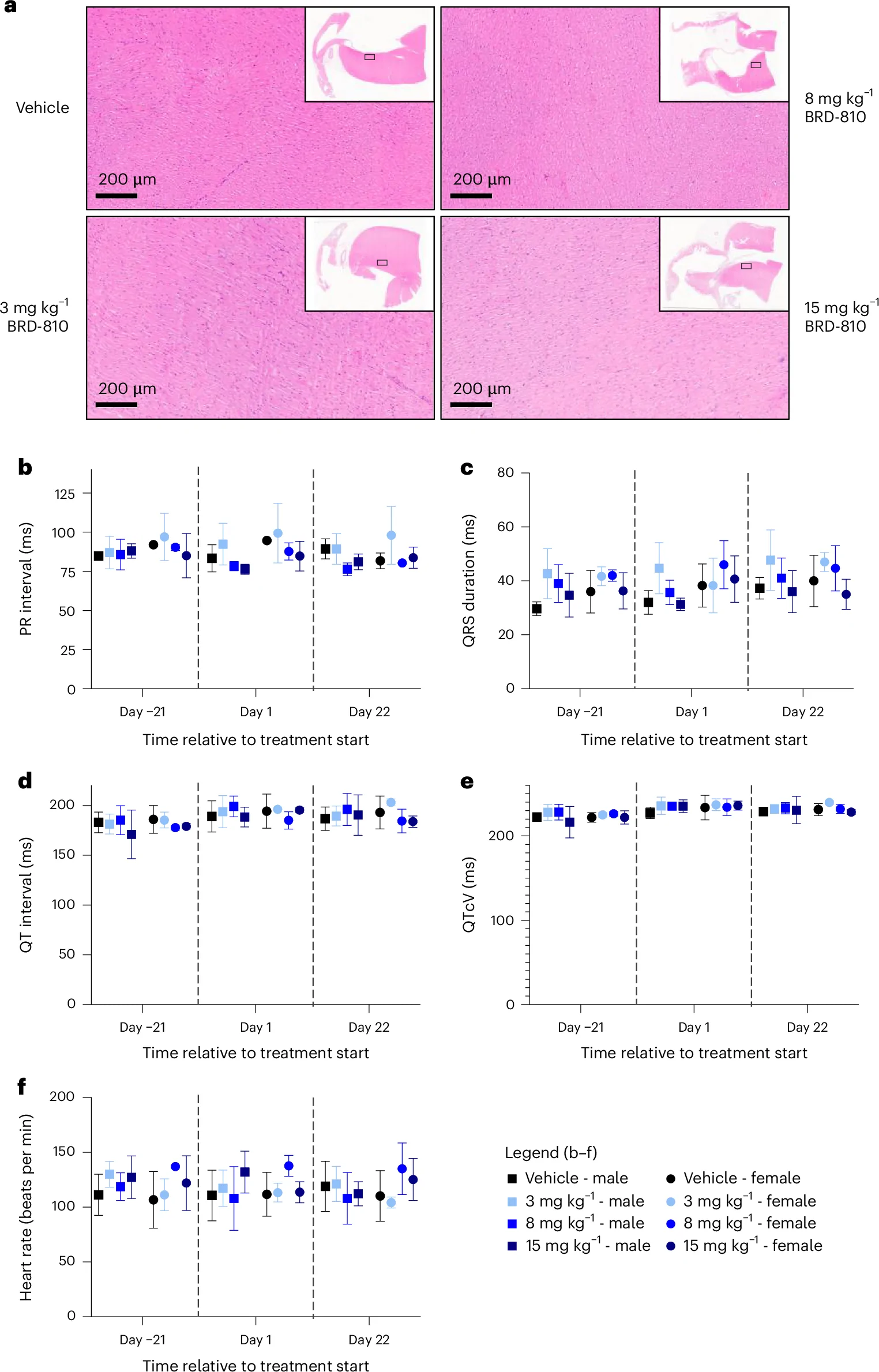
Histopathology analysis of heart tissue and ECG analysis from dogs repeatedly treated with BRD-810. **a**, Beagle dogs were intravenously infused with BRD-810 for 4 h once weekly for 4 weeks before heart tissue was prepared. One representative picture is shown per group (*n* = 6 beagle dogs per group; three female and three male). **b**–**f**, ECG parameters (**b**–**e**) and heart rate (**f**) of beagle dogs before treatment (day −21), at the start of treatment (day 1, within 10 min of ceasing dosing) and after the fourth dose (day 22, within 10 min of ceasing dosing). Data are shown as the mean and s.d. (*n* = 3 beagle dogs per group and gender). Source data

In addition to cardiac troponin measurements, we performed an ECG to demonstrate that repeat dosing of BRD-810 had no impact on key ECG parameters (PR, QRS, QT and calculated QTcV intervals) or heart rate (Fig. 6b–f). In addition, we analyzed heart tissue from all dogs following necropsy at the end of the 4-week repeat-dose study (Fig. 6a). In alignment with the absence of increased troponin I levels in the blood, histopathological analysis of heart tissue showed no evidence of cardiac tissue injury following BRD-810 treatment. Overall, the absence of cardiotoxicity with short-term infusion of BRD-810 suggests that its unique pharmacokinetic profile is suitable for clinical investigation.

## Discussion

Amplification of the *MCL1* locus is one of the most frequent somatic genetic events in human cancer^2^ and overexpression of *MCL1* in response to chemotherapy or targeted agents is a frequent cause of cancer resistance to current treatments^22,25,29^. However, given recent reports of cardiac safety signals during the early clinical development of MCL1 inhibitors and the emerging biology of MCL1 in normal cardiomyocyte function^45^, the key question is whether inhibition of MCL1 is safe to pursue for the treatment of cancer.

Here, we introduce BRD-810, a rapidly cleared inhibitor of MCL1 that has robust antitumor efficacy in hematological and solid tumor cancer models. We show that BRD-810 binds to the BH3 groove of MCL1 and blocks sequestration of proapoptotic proteins to rapidly induce caspase activation in MCL1-dependent cell lines with superior potency when compared to other clinical-stage MCL1 inhibitors. BRD-810 induced cancer cell death in a large panel of hematologic and solid tumor cell lines, reflecting its broad potential to promote apoptosis even in treatment-refractory settings. While hematologic cancer cell lines were on average more sensitive to BRD-810 treatment than solid tumor cells, sensitivity to BRD-810 was observed across tumor indications and we propose the ratio of *BCL2L1* to *BAK* mRNA as a potential biomarker for patient stratification in ongoing and future MCL1 inhibitor trials. This biomarker profile is in line with earlier studies and resonates with the understanding that Bcl-X_L_ can compensate for MCL1 loss while BAK is a proapoptotic protein required to induce the intrinsic apoptotic pathway^41,57,58^.

Importantly, through time-course and washout experiments, we established that caspase activation occurs within hours of BRD-810 exposure, initiating an irreversible process of outer mitochondrial membrane permeabilization that ultimately leads to cell death. The rapid cell-killing effects of BRD-810 were confirmed in several in vivo tumor models. Despite a short residence time in plasma, a single bolus injection of BRD-810 sufficiently overwhelmed the antiapoptotic threshold in in vivo models of hematological and solid cancers and led to measurable tumor volume reduction as early as within the first 24 h. Because apoptosis is irreversible, prolonged exposure to MCL1 inhibitors is not required for effective tumor reduction.

Transient MCL1 inhibition creates an opportunity to minimize the potential on-target toxicity of MCL1-targeting therapies. To model the effects of BRD-810 on cardiomyocyte viability, we investigated 4-h and continuous exposure of BRD-810 to hiPS cell-derived cardiomyocytes and found that undesirable effects on LDH and troponin release were prevented with shorter BRD-810 incubation. Our data suggest that 4 h of MCL1 inhibition is sufficient to push MCL1-dependent cells down the irreversible path of apoptosis without severely compromising the viability of other cells, including cardiomyocytes. Therefore, an MCL1 inhibitor such as BRD-810, which is rapidly cleared from systemic circulation, may offer an opportunity to test this hypothesis in humans.

## Methods

All research performed complied with the ethical regulations of the regions where the studies were performed. All mouse studies were performed under German and European animal welfare regulations and approved by local authorities (LAGeSo). All dog studies were performed in accordance with the Organization for Economic Co-operation and Development (OECD) principles of good laboratory practice as accepted by regulatory authorities throughout the European Union, USA (Food and Drug Administration (FDA) and Environmental Protection Agency) and Japan (Ministries of Health, Labor and Welfare, Agriculture, Forestry and Fisheries and Economy, Trade and Industry), as well as other countries that are signatories to the OECD Mutual Acceptance of Data agreement.

### Proteins, BH3-derived peptides and compounds

MCL1 protein (amino acids 173–321), used for in vitro biological assays (surface plasmon resonance (SPR), HTRF and X-ray crystallography), was expressed and purified as previously described using a bacterial expression system^49^. Bcl-2 and Bcl-X_L_ protein for HTRF assays was purchased from BPS Bioscience. BH3-derived peptides (for example, Noxa and Bad) were purchased from Biosyntan with the following sequences: Noxa, biotin-PEG2-PEG2-PAELEVE-Nva-ATQLRRFGDKLNFRQKLL-amide; Bad, biotin-PEG2-PEG2-NLWAAQRYGRELRR-Nle-SDEFVDSFKK-amide. Compounds **1** and **2** and BRD-810 were synthesized as described in WO 2017198341 (compound **1**), WO 2019096905 (compound **2**) and WO 2020236556 (BRD-810). The MCL1 inhibitors AZD5991 (ref. ^55^), AMG176 (ref. ^41^) and S64315 (ref. ^43^) were made in house according to published methods. Etoposide and cisplatin were purchased from Selleckchem.

### Biochemical and biophysical assays

Time-resolved fluorescence energy transfer (TR-FRET) assays were used to measure BRD-810-mediated dose-dependent inhibition of MCL1 and Noxa (BH3-derived peptide) binding as previously described in detail (reference patent). Briefly, MCL1 protein served as the protein receptor and Noxa served as the tracer ligand, a compound dose range of 0.1–20,000 nM was tested in duplicate across the same microtiter plate and the assay was initiated by the addition of 2 µl of a 2.5-fold concentrated MCL1 protein solution (1 nM final concentration) in aqueous assay buffer to the compounds in the assay plate. The fluorescence emission at 620 nm and 665 nm after excitation at 330–350 nm was measured using Rubystar (BMG Lab Technologies), Pherastar (BMG Lab Technologies) or Viewlux (PerkinElmer) plate readers. IC_50_ values were determined by regression analysis based on a four-parameter (4-PL) equation (minimum, maximum, IC_50_ and Hill; *y* = max + (min − max)/(1 + (*x*/IC_50_) Hill) using the Screener Software (Genedata). For selectivity testing with Bcl-X_L_ and Bcl-2, similar protocols were used with the following modifications: Bcl-X_L_ protein receptor with BAD peptide as the tracer ligand or Bcl-2 protein receptor with BAD peptide as the tracer ligand.

For SPR assays measuring BRD-810 binding to MCL1 protein, Biacore T200 or S200 (Cytiva Life Sciences) instruments were used as previously described (patent WO 2019096922). For SPR measurements, MCL1 protein was immobilized using standard amine coupling and serial dilutions of compound (eight dilution steps, typically ranging from 0.2 nM up to 1,000 nM) were injected over immobilized protein. The double-referenced sensorgrams were fit to a simple reversible Langmuir 1:1 reaction mechanism as implemented in the Biacore T200 and S200 evaluation software.

### Cocrystallization of MBP–MCL1 bound to BRD-810

Crystallization was accomplished using our previously reported method^47^ with several modifications to provide a rapid, reliable crystallization system with improved cocrystallization success rates for low-solubility compounds. Briefly, 2.5 μl of solubilized BRD-810 solution (1 mM in 20% PEG400, 40% methyl prednisolone and 40% DMSO) was placed on the side of a thin-walled PCR tube. To improve compound solubilization, this drop was rapidly flushed with 7.5 μl of maltodextrin-binding protein (MBP)–MCL1 protein (10 mg ml^−1^ in 2 mM maltose, 20 mM HEPES pH 7.5, 200 mM NaCl, 1% glycerol and 2 mM DTT), mixed thoroughly and allowed to incubate for 15 min on ice. Then, 3 μl of this protein–compound mix was combined with 3 μl of precipitant (seed) solution (16% PEG3350, 50 mM magnesium formate, 20 mM HEPES pH 7.5 and 1:10,000 diluted microseeds) in an EasyXtal 15-well DropGuard crystallization tool. This completed mixture was sealed and allowed to equilibrate against 400 μl of 1.5 M NaCl. Protein crystals appeared within 1 day and continued to grow for approximately 7 days. A single crystal with dimensions of approximately 40 μM × 80 μM × 200 μM was then harvested directly from the crystallization drop in a nylon cryoloop and flash-frozen by rapidly plunging into liquid nitrogen. X-ray diffraction datasets were collected from frozen single crystals at the ALBA synchrotron and processed and refined with the programs xia2 and DIALS from the CCP4 program suites^59–61^ (Supplementary Table 2). Automated refinement and ligand identification were performed using DIMPLE and a known MBP–MCL1 protein as a model (Protein Data Bank (PDB) 4WGI). Iterative model rebuilding and refinement were performed using the programs Coot and Phenix^62,63^. Figures were generated using PyMOL and MOE. All software used for data processing, refinement and modeling were accessed using SBGrid^64^.

### Cell lines

The following cell lines were used in this study: RKO (colon carcinoma; American Type Culture Collection (ATCC), CRL-2577), HMC1-8 (breast cancer; Japanese Collection of Research Bioresources, JCRB0166), MV-4-11 (AML; ATCC, CRL-9591), THP-1 (AMP; ATCC, TIB-202), MOLP-8 (multiple myeloma; DMSZ, ACC569), AMO-1 (multiple myeloma; German Collection of Microorganisms and Cell Cultures (DSMZ), ACC538), SU-DHL-4 (DLBCL; DSMZ, ACC495), SU-DHL-5 (DLBCL; DSMZ, ACC571), SU-DHL-10 (DLBCL; DSMZ, ACC576), KMS-12-BM (multiple myeloma; DSMZ, ACC551), RPMI 8226 (multiple myeloma; DSMZ, ACC402), OPM-2 (multiple myeloma; DSMZ, ACC50), JJN-3 (plasma cell leukemia; DSMZ, ACC541), KMS-12-PE (multiple myeloma; DSMZ, ACC606), DMS114 (lung cancer; ATCC, CRL2066), A-427 (lung cancer; ATCC, HT-B53), HCC-1187 (breast cancer; ATCC, CRL-2322), SNU398 (liver cancer; ATCC, CRL-2233), HCC-2157 (breast cancer; ATCC, CRL-2340), PA-1 (ovarian cancer; ATCC, CRL-1572), NCI-H82 (lung cancer; ATCC, HTB-175), SNU-16 (gastric cancer; ATCC, CRL-5974) and A-431 (melanoma; ATCC, CRL-1555). Authentication of cell lines was performed using karyotyping and PCR analysis with species-specific primers. Authentication was performed together with *Mycoplasma* testing before cell stocks were frozen. Cell stocks were used for a maximum of 3 months before a fresh vial was thawed.

### Cellular assays

*BAK*;*BAX* double-knockout cells were generated using the CRISPR–Cas9 system. Briefly, HMC1-8 cells were infected with lentivirus made from pLX-311-Cas9-Blast plasmid and packaging plasmid (Genetic Perturbation Platform (GPP), Broad Institute) to establish a stable cell line expressing Cas9. Blasticidin (5 μg ml^−1^) was used for positive selection. Then, those cells were infected with lentivirus made from packaging plasmids and a single guide RNA (sgRNA) vector (pXPR-003, GPP, Broad Institute) containing an sgRNA sequence (*BAK*, CACCGGTTGATGTCGTCCCCGATGA; *BAX*, CACCGAGCGAGTGTCTCAAGCGCAT). Virus production and infection were based on the Broad Institute GPP protocols (https://portals.broadinstitute.org/gpp/public/resources/protocols). Pooled cells were used in the viability and caspase activity experiments.

The hiPS cell-derived cardiomyocytes used in this study were commercially available (Ncyte, Ncardia, cat. no. Nc-C-BRCM). They were thawed and cultured according to Ncardia’s instruction manual and maintained with Ncardia’s proprietary cardiomyocyte culture medium (Ncardia, cat. no. Nc-M-CMCM-250). The cells were seeded at a density of 15,000 cells per well in 96-well plates for all subsequent assays. BRD-810 and doxorubicin (Selleckchem, cat. no. S1208) were dissolved as 1 mM and 10 mM stock concentrations in DMSO, respectively, and were diluted accordingly to the desired test concentrations used in the cells (0.1 and 1 μM for BRD-810 and 10 μM for doxorubicin). Compounds were added 7 days after seeding the cells and the DMSO solvent did not exceed 0.1% (v/v) for each well. The 96-well plates with cells were maintained in an incubator at 37 °C in 5% CO_2_ for the duration of the experiment.

Immunoprecipitation and western blot analysis were performed as previously described using antibodies for Mcl1 (Cell Signaling Technology, clone D35A5, cat. no. 5453; 1:1,000), BAK (Cell Signaling Technology, clone D2D3, cat. no. 6947; 1:1,000) and Bim (Cell Signaling Technology, clone C34C5, cat. no. 2933; 1:1,000)^65^. The activity of caspases 3 and 7 was determined in multiple cell lines (SU-DHL-4, SU-DHL-5, SU-DHL-10, HMC1-8 parental, HMC1-8 Cas9 control, and HMC1-8 *BAK*;*BAX* knockout) upon treatment with different compounds using the Caspase-Glo 3/7 reagent from Promega (cat. no. G8092). Cell lines were plated in culture medium (RPMI 1640; Gibco, cat. no. 22400-089) supplemented with 10% FBS at a density of 3,300 cells in 30 pl per well in a sterile, solid, black, flat-bottom polystyrene tissue culture (TC)-treated 384-well microplate (Corning, cat. no. 3571) using a Multidrop Combi reagent dispenser. As a control, medium without cells was also added to the plate. Cells were incubated in a humidified incubator at 37 °C overnight. On the next day, the cells were treated with compounds (stock solution, 10 mM in DMSO) using the HP D300 digital dispenser in a concentration range of 3.3 × 10^−5^ M (33,000 nM) to 5 × 10^−9^ M (5 nM) in a single-dot curve with at least 16 dilutions and a DMSO concentration of 0.33%. Rim wells were excluded. The cells were incubated for 3 h in a humidified incubator at 37 °C. After this incubation, 30 pl of Caspase-Glo 3/7 reagent (Promega, cat. no. G8092) was added to each well using the Multidrop Combi reagent dispenser, followed by 1-h incubation at 37 °C. Finally, luminescence was read at 0.1 ms with a gain of 3,000 using the PHERAstar FS microplate reader (BMG Labtech).

Gene expression was quantified in a breast cancer cell panel using qPCR. Total RNA was extracted using the RNeasy Mini kit (Qiagen) and complementary DNA was generated using the SuperScript III first-strand synthesis kit (Thermo Fisher). TaqMan probe-based qPCR (Thermo Fisher) was carried out using a TaqMan fast advanced master mix and TapMan primer + probe sets for *BCL2L1* Hs00236329_m1 and *BAK* Hs00832876_g1 (both from Thermo Fisher). qPCR was measured on a LightCycler 480 (Roche) real-time PCR system. Gene expression was normalized to *GAPDH* expression levels measured with the primer + probe set Hs02786624_g1 (Thermo Fisher).

### Cell viability assays

The impact of compounds on the proliferation of different cell lines was assessed using the CellTiter-Glo luminescent cell viability reagent from Promega (cat. no. G7573). The different cell lines were plated in culture medium (RPMI 1640; Biochrom, cat. no. FG 1215]) supplemented with 10% FCS (Biochrom, cat. no. S 0415) at a density of 3,300 cells (for suspension cells) or 800 cells (for adherent cells) in 30 pl per well in a sterile, solid, black, flat-bottom polystyrene TC-treated 384-well microplate (Corning, cat. no. 3571) using a Multidrop Combi reagent dispenser. In parallel, cells were plated in a reference (day 0) plate for time zero determination. Cells were incubated in a humidified incubator at 37 °C overnight. On the next day, cells were treated with compounds (stock solution, 10 mM in DMSO) using the HP D300 digital dispenser in a concentration range of 3.3 × 10^−5^ M (33 µM) to 5 × 10^−9^ M (5 nM) in a single-dot curve with at least 16 dilutions and a DMSO concentration of 0.33%. Rim wells were excluded. The cells were incubated for 72 h in a humidified incubator at 37 °C. The day-0 plate was measured by adding 30 pl per well of CellTiter-Glo luminescent cell viability reagent (Promega, cat. no. G7573) to time zero wells in the reference plate followed by a 10-min incubation and luminescence reading at 0.1 ms using the PHERAstar FS microplate reader (BMG Labtech).

For viability evaluation according to nuclear count (high-content imager), cells were fixed with 4% PFA (Thermo Fisher, cat. no. 28908) and were stained with DAPI dye. Following DAPI staining, each plate was imaged with an ImageXpress Micro confocal high-content imaging system (Molecular Devices). Using the MetaXpress image analysis software (Molecular Devices), the number of nuclei per well was calculated for all wells. Cell viability was expressed as the number of nuclei in the well divided by the average number of nuclei of vehicle control (DMSO). Data from three replicate wells of each condition were averaged. Bar graphs represent the average and error bars represent the s.d. of the averages.

The levels of extracellular LDH were evaluated using the commercially available LDH Glo assay (Promega, cat. no. J2380). The assay was performed according to the manufacturer’s instructions and luminescence was recorded using a CLARIOstar (BMG) plate reader.

### ELISA assay for MCL1–BAK or MCL1–BIM interaction

On day 1, RKO colon cancer cell line cells were plated at 0.8 million cells per ml (100 µl per well) in 96-well flat-bottom TC-treated plates (Corning). MCL1 antibody (Santa Cruz, sc-12756) was diluted 200-fold (final concentration, 1 µg ml^−1^) in carbonate buffer (pH 9.6; Thermo Fisher) and 50 µl of diluted antibody was added to each well of high-bind ELISA plates (SARSTEDT). Each plate was tapped to make sure liquid covered the entire bottom of the wells and then incubated at 37 °C overnight. On the second day, MCL1 antibody was washed from the ELISA plate. Then, 250 µl of Odyssey blocking buffer (PBS) (Li-Cor) was added to each well, incubated at room temperature for at least 1 h and then washed once with 250 µl of 1× PBST. BRD-810 was added to plates with RKO cells using a HP Tecan D300e compound dispenser in a threefold dilution series (highest dose 30 µM), with ten doses per compound in quadruplicate. After incubation at 37 °C for the indicated times, plates with RKO cells were gently washed once with 100 µl of PBS per well and then 50 µl of CHAPS buffer (50 mM Tris-CI pH 7.4, 150 mM NaCl, 1% CHAPS, 1 mM EDTA, 1 mM EGTA, complete protease inhibitors (Roche) and PhosSTOP (Roche)) was added to each well. Plates were rocked for 1 h at 4 °C and then 45 µl of cell lysate from each well was transferred to ELISA plates coated with MCL1 antibody. Plates were incubated overnight in the cold room with rocking. On the third day, ELISA plates were washed once with 250 µl of 1× PBST. Biotinylated anti-BAK (Santa Cruz, sc-1035) or anti-Bim (Cell Signaling, 2933) antibody was added and incubated for 2 h. Wells were washed once with 250 µl of 1× PBST. Streptavidin poly(horseradish peroxidase) (Thermo Fisher) was diluted to 20 ng ml^−1^ in Odyssey blocking buffer plus 0.05% Triton X-100 and 100 µl was added to each well of the ELISA plate. Plates were incubated at room temperature for 1 h with rocking and then washed with 100 µl of 1× PBST three times. Each SuperSignal ELISA Femto maximum-sensitivity substrate (Thermo Fisher) was added to a 50-ml tube and mixed and then 100 µl of mixed substrate was added to each well. Plates were shaken for 1 min and then luminescence was measured using an Envision plate reader (HP). The signal of each well was normalized by the no-compound control and no-cell control. The IC_50_ was calculated using GraphPad Prism software.

### Bioinformatic analysis

To identify a subset of baseline genomic features of the cell lines that best explained the measured MCL1 inhibitor sensitivity (area under the curve (AUC)), a variational approximation to a Bayesian linear regression variable selection model was used, implemented in the ‘varbvs’ R package^66^. Five different values for the ‘logodds’ hyperparameter were used, ranging from 10^−5^ to 10^−1^ and were shown as the posterior mean regression coefficients plotted against the Pearson correlation between each feature and the AUC.

### In vivo safety studies in dog

All dog studies were performed in accordance with the OECD principles of good laboratory practice as accepted by regulatory authorities. Purpose-bred, naive beagle dogs (6–9 months old; Supplier Marshall UK for Charles River) were provided with an intravenous port and were individually fitted with a custom-designed dog jacket, including a protective collar (if necessary), and acclimatized to them for a minimum of 5 days before the treatment period started. Dogs were randomized into four groups: vehicle group (five male and five female), 3 mg kg^−1^ BRD-810 group (three male and three female), 8 mg kg^−1^ BRD-810 group (three male and three female) and 15 mg kg^−1^ BRD-810 group (five male and five female). During the treatment period, BRD-810 was infused once weekly at 3, 8 or 15 mg kg^−1^ for 4 weeks. At the end of the 4-week treatment period, two male and two female dogs from the vehicle group and the 15 mg kg^−1^ group were admitted to the recovery period. Full toxicology analysis was performed including cardiac toxicity evaluation.

### Cardiac toxicity evaluation

ECGs were measured using a Cardiofax ECG 9620 electrocardiograph (Nihon Kohden Europe). Heart tissue for histopathology was collected at the end of the study and embedded in paraffin, sectioned, mounted on glass slides, stained with hematoxylin and eosin and evaluated histopathologically by a board-certified veterinary pathologist blinded to treatment groups.

For the troponin I assay, supernatants (50 μl per well) were collected from each well of the 96-well plates in culture and were immediately frozen at −80 °C. On the day of the assay, the supernatant samples were thawed at room temperature and were used to perform the troponin I AlphaLISA assay (PerkinElmer, cat. no. CUSM86458000EASBR1) according to the manufacturer’s instructions. The standard curve of the assay was generated using a nonlinear regression 4-PL sigmoidal curve fit and the LLOD (lower level of detection) was calculated at 1.8 pg ml^−1^ cTnI. Positive control and test samples with a notable cardiotoxic effect (exceeding the 1,000 pg ml^−1^ threshold value) were found within the dynamic range of the assay.

### In vivo efficacy and assays

BRD-810 efficacy was tested in xenograft models of multiple myeloma (MOLP-8 and AMO-1), DLBCL (SUD-HL-10), liver cancer (SNU398), lung cancer (A-427) and AML (THP-1). Human tumor cells (5–10 × 10^6^ suspended in Matrigel) of the respective cancer type were injected subcutaneously into 6–8-week-old immunocompromised mice (female CB17/SCID mice (C.B-*Igh-1*^*b*^/IcrTac-*Prkdc*^*scid*^), Taconic). Once the primary tumor growth was established (>30 mm^2^) animals were randomized and assigned to study groups. Animals received intravenous treatment with BRD-810 at the indicated doses and schedules or vehicle control. The treatment response to BRD-810 versus control groups was assessed by a determination of tumor area using a caliper. Animals were killed once they were close to reaching the maximum bearable tumor size of 250 mm^2^. The tumor size was not exceeded. The principles of such xenograft studies were previously summarized^67^.

For in vivo caspase 3 measurements in the MOLP-8 model, tumors were sampled from mice at different time points following a single intravenous dose of BRD-810 and protein was extracted. Western blot analysis was performed using human-specific antibodies for total caspase 3 (Cell Signaling Technology, clone 3G2, cat. no. 9668; 1:1,000) and cleaved caspase 3 (Abcam, clone E83-77, cat. no. ab32042; 1:1,000). Western blot bands were quantified using densitometric analysis.

### Statistics and reproducibility

No statistical methods were used to predetermine sample sizes but our sample sizes are similar to those reported in previous publications^41,55^. Sample sizes for dog studies were chosen according to the FDA International Council for Harmonization of Technical Requirements for Pharmaceuticals for Human Use S9 guideline. In vitro experiments were performed with at least three biological replicates and repeated in independent experiments. All attempts at replication were successful. For in vivo studies, mice were randomized according to the ‘tumor size matched distribution’ method before the start of treatment to ensure that each group started with an approximately equal mean tumor size. Male and female beagle dogs were randomized separately to achieve similar group mean body weights while ensuring that litter mates were homogeneously distributed across all groups where possible. The person performing histopathology analyses was blinded to the study. The persons performing the in vivo mice studies were blinded to the expected outcome and mode of action of the compounds tested. For all other studies, data collection and analysis were not performed blind to the conditions of the experiments. No data were excluded from the analyses. Statistics were performed on values from independent experiments. All statistical calculations were performed with GraphPad Prism 10. Data distribution was assumed to be normal but this was not formally tested. Error bars represent the s.d. Box plots are shown with the median and quartiles as described in the figure legends.

### Reporting summary

Further information on research design is available in the Nature Portfolio Reporting Summary linked to this article.

## Supplementary information

Reporting Summary

Supplementary Tables Supplementary Tables 1–5.

Supplementary Data Validation report.

## Source data

Source Data Fig. 1 Statistical source data.

Source Data Fig. 2 Statistical source data.

Source Data Fig. 3 Statistical source data.

Source Data Fig. 4 Statistical source data.

Source Data Fig. 5 Statistical source data.

Source Data Fig. 6 Statistical source data.

Source Data Fig. 6 Unprocessed, high-resolution histopathology data.

Source Data Extended Data Figs. 3 and 4 Statistical source data.

## Data Availability

Structural data for MBP–MCL1 bound to BRD-810 can be accessed through the PDB database under accession code 8T6F. All other data that support the findings of this study are available from the corresponding authors upon request. Source data are provided with this paper.
