# Supplementary material for: BRD-810 is a highly selective MCL1 inhibitor with optimized in vivo clearance and robust efficacy in solid and hematological tumor models
Source: Nat Cancer. 2024 Aug 23;5(10):1479–93. doi: 10.1038/s43018-024-00814-0 (PMC11502502; doi:10.1038/s43018-024-00814-0)
Supplement: Supplementary file 1 — Reporting Summary [file 43018_2024_814_MOESM1_ESM.pdf]

nature portfolio

Corresponding author(s): Ulrike Rauh

Last updated by author(s): Jul 22, 2024

Reporting Summary

Nature Portfolio wishes to improve the reproducibility of the work that we publish. This form provides structure for consistency and transparency in reporting. For further information on Nature Portfolio policies, see our Editorial Policies and the Editorial Policy Checklist.

Statistics

For all statistical analyses, confirm that the following items are present in the figure legend, table legend, main text, or Methods section.

n/a	Confirmed	
☐	☒	The exact sample size (*n*) for each experimental group/condition, given as a discrete number and unit of measurement
☐	☒	A statement on whether measurements were taken from distinct samples or whether the same sample was measured repeatedly
☒	☐	The statistical test(s) used AND whether they are one- or two-sided<br>*Only common tests should be described solely by name; describe more complex techniques in the Methods section.*
☒	☐	A description of all covariates tested
☒	☐	A description of any assumptions or corrections, such as tests of normality and adjustment for multiple comparisons
☐	☒	A full description of the statistical parameters including central tendency (e.g. means) or other basic estimates (e.g. regression coefficient) AND variation (e.g. standard deviation) or associated estimates of uncertainty (e.g. confidence intervals)
☐	☒	For null hypothesis testing, the test statistic (e.g. *F*, *t*, *r*) with confidence intervals, effect sizes, degrees of freedom and *P* value noted<br>*Give P values as exact values whenever suitable.*
☐	☒	For Bayesian analysis, information on the choice of priors and Markov chain Monte Carlo settings
☒	☐	For hierarchical and complex designs, identification of the appropriate level for tests and full reporting of outcomes
☐	☒	Estimates of effect sizes (e.g. Cohen's *d*, Pearson's *r*), indicating how they were calculated

Our web collection on statistics for biologists contains articles on many of the points above.

Software and code

Policy information about availability of computer code

Data collection	*Provide a description of all commercial, open source and custom code used to collect the data in this study, specifying the version used OR state that no software was used.*
Data analysis	XAI2 version 3.8.6, DIALS version 3.8, CCP4 version 8.0, DIMPLE version 2.6.2, COOT version 0.9.8.3, Phenix version 1.20.1, Pymol version 2.5.5, SBGrid version 2.9.9, MOE version 2022.02, Graph Pad PRISM 10 version 10.0.0 (153)

For manuscripts utilizing custom algorithms or software that are central to the research but not yet described in published literature, software must be made available to editors and reviewers. We strongly encourage code deposition in a community repository (e.g. GitHub). See the Nature Portfolio guidelines for submitting code & software for further information.

Data

Policy information about availability of data

All manuscripts must include a data availability statement. This statement should provide the following information, where applicable:

- Accession codes, unique identifiers, or web links for publicly available datasets
- A description of any restrictions on data availability
- For clinical datasets or third party data, please ensure that the statement adheres to our policy

Structural data MBP-MCL1 bound to BRD-810 (PDB 8T6F) can be accessed through the PDB database under the code PDB 8T6F. Source data have been provided as source data files. All other data that support the findings of this study are available from the corresponding author upon request.

Human research participants

Policy information about studies involving human research participants and Sex and Gender in Research.

Reporting on sex and gender	N/A
Population characteristics	N/A
Recruitment	N/A
Ethics oversight	N/A

Note that full information on the approval of the study protocol must also be provided in the manuscript.

Field-specific reporting

Please select the one below that is the best fit for your research. If you are not sure, read the appropriate sections before making your selection.

☒ Life sciences ☐ Behavioural & social sciences ☐ Ecological, evolutionary & environmental sciences

For a reference copy of the document with all sections, see nature.com/documents/nr-reporting-summary-flat.pdf

Life sciences study design

All studies must disclose on these points even when the disclosure is negative.

Sample size	No statistical methods were used to pre-determine sample size, but our sample sizes are similar to those reported in previous publications: PMID:30254093, PMID:30559424. Sample size for dog studies were chosen according to the FDA ICH S9 guideline.
Data exclusions	no data was excluded
Replication	In vitro experiments were performed at least with 3 biological replicates and repeated in independent experiments. All attempts at replication were successful. in vivo studies were performed only once with appropriate individual sample sizes.
Randomization	Randomization for mice studies was performed based on 'tumor size matched distribution' method before treatment start. For dog studies, male and female Beagle dogs were randomized separately to achieve similar group mean body weights, while ensuring that litter mate were homogenously distributed across all groups, where possible
Blinding	The person performin histopathology analyses was blinded to the study. The persons performing the in vivo studies were blinded to the expected outcome and mode-of-action of the compounds tested. For all other studies data collection and analysis were not performed blind to the conditions of the experiment.

Reporting for specific materials, systems and methods

We require information from authors about some types of materials, experimental systems and methods used in many studies. Here, indicate whether each material, system or method listed is relevant to your study. If you are not sure if a list item applies to your research, read the appropriate section before selecting a response.

Materials & experimental systems

n/a	Involved in the study	
☐	☒	Antibodies
☐	☒	Eukaryotic cell lines
☒	☐	Palaeontology and archaeology
☐	☒	Animals and other organisms
☒	☐	Clinical data
☒	☐	Dual use research of concern

Methods

n/a	Involved in the study	
☒	☐	ChIP-seq
☒	☐	Flow cytometry
☒	☐	MRI-based neuroimaging

Antibodies

Antibodies used	Western Blot: Mcl-1, Cell Signaling Technology, clone D35A5, catalog#5453, dilution 1:1000; BAK, Cell Signaling Technology, clone D2D3, catalog #6947, dilution 1:1000: Bim, Cell Signaling Technology, clone C34C5; catalog #2933, dilution 1:1000; total caspase-3,

	Cell Signaling Technology, Clone 3G2, catalog #9668, 1:1000, cleaved caspase-3 Abcam, clone E83-77, catalog # ab32042, dilution 1:1000, Co-immunoprecipitation MCL1 antibody, Santa Cruz, clone 22, catalog# sc-12756, 1µM per IP
Validation	MCL1 antibody, Santa Cruz sc-12756 were validated in PMID: 22516262, validation for Mcl-1 antibody, Cell Signaling Technology, clone D35A5, calatog#5453, can be found here: https://www.cellsignal.com/products/primary-antibodies/mcl-1-d35a5-rabbit-mab/5453; validation for BAK-antibody, Cell Signaling Technology, clone D2D3, catalog #6947 can be found here: https://www.cellsignal.com/products/primary-antibodies/bak-d2d3-rabbit-mab/6947and for Bim-Antibody, Cell Signaling Technology, clone C34C5, catalog #2933 can be found here https://www.cellsignal.com/products/primary-antibodies/bim-c34c5-rabbit-mab/2933

Eukaryotic cell lines

Policy information about cell lines and Sex and Gender in Research

Cell line source(s)	RKO, Colon carcinoma, ATCC, CRL-2577; HMC-1-8, breast cancer, JCRB0166; MV-4-11 (AML, ATCC, CRL-9591); THP-1 (AMP, ATCC, TIB-202), MOLP-8 (Multiple Myeloma, DMSZ, ACC569); AMO-1 (Multiple Myeloma, DSMZ, ACC538); SU-DHL-4 (DLBCL, DSMZ, ACC495), SU-DHL-5 (DLBCL, DSMZ, ACC571), SU-DHL-10 (DLBCL, DSMZ, ACC576), KMS-12-BM (Multiple Myeloma, DSMZ, ACC551), RPMI8226 (multiple myeloma, DSMZ, ACC402), OPM-2 (Multiple myeloma, DSMZ, ACC50), JJN-3 (Plasma cell leukemia, DSMZ, ACC541), KMS-12-PE (Multiple myeloma, DSMZ, ACC606), DMS114 (lung cancer, ATCC, CRL2066), A-427 (lung cancer, ATCC, HT-B53), HCC-1187 (breast cancer, ATCC, CRL-2322), SNU398 (liver cancer, ATCC, CRL-2233), HCC-2157 (breast cancer, ATCC, CRL-2340), PA-1 (ovarian cancer, ATCC-CRL-1572), NCI-H82 (lung cancer, ATCC, HTB-175), SNU-16 (gastric cancer, ATCC, CRL-5974), A-431 (melanoma, ATCC, CRL-1555)
Authentication	Authentication was performed using Karyotyping and PCR analysis using species-specific primers before cell stocks were frozen.
Mycoplasma contamination	Mycoplasma tests were performed before cell stocks were frozen. Only Mycoplasma negative cell lines were stored in stock
Commonly misidentified lines (See ICLAC register)	None of the ICLAC misidentified cell lines was used in this study

Animals and other research organisms

Policy information about studies involving animals; ARRIVE guidelines recommended for reporting animal research, and Sex and Gender in Research

Laboratory animals	Mice studies were performed with 6-8weeks old female CB17/SCID mice [C.B-Igh-1b/IcrTac-Prkdcscid], Taconic ; Dog studies were performed with 6-9 months old female and male Beagle Dogs (Supplier Marshall UK for Charles River)
Wild animals	No wild animals were used in this study.
Reporting on sex	Mice used in this study were female to enable housing requirements, tumors tested were of female and male human origin, for dogs both genders were tested
Field-collected samples	This study did not involve field-collected samples.
Ethics oversight	For mouse tumor studies the maximum allowed tumor size of 250 mm2 was not exceeded in any of the studies. All animal studies were performed under German and European Animal Welfare Regulations and approved by local authorities (LAGESO, Berlin, Germany).All dog studies were performed in accordance with the OECD Principles of Good Laboratory Practice as accepted by Regulatory Authorities throughout the European Union, United States of America (FDA and EPA) and Japan (MHLW, MAFF and METI) and other countries that are signatories to the OECD Mutual Acceptance of Data Agreement.

Note that full information on the approval of the study protocol must also be provided in the manuscript.
