# Supplementary material for: BRD-810 is a highly selective MCL1 inhibitor with optimized in vivo clearance and robust efficacy in solid and hematological tumor models
Source: Nat Cancer. 2024 Aug 23;5(10):1479–93. doi: 10.1038/s43018-024-00814-0 (PMC11502502; doi:10.1038/s43018-024-00814-0)
Supplement: Supplementary file 3 — Validation report. [file 43018_2024_814_MOESM3_ESM.pdf]

Full wwPDB X-ray Structure Validation Report (i)

Aug 1, 2023 – 02:28 PM EDT

PDB ID	:	8T6F
Title	:	Crystal structure of human MBP-Myeloid cell leukemia 1 (Mcl-1) in complex with BRD810 inhibitor.
Deposited on	:	2023-06-15
Resolution	:	1.56 Å(reported)

This wwPDB validation report is for manuscript review

This is a Full wwPDB X-ray Structure Validation Report.

This report is produced by the wwPDB biocuration pipeline after annotation of the structure.

We welcome your comments at *validation@mail.wwpdb.org*
A user guide is available at
https://www.wwpdb.org/validation/2017/XrayValidationReportHelp
with specific help available everywhere you see the (i) symbol.

The types of validation reports are described at
http://www.wwpdb.org/validation/2017/FAQs#types.

For Manuscript Review

The following versions of software and data (see references (i)) were used in the production of this report:

MolProbity	:	4.02b-467
Mogul	:	1.8.5 (274361), CSD as541be (2020)
Xtriage (Phenix)	:	1.13
EDS	:	2.34
buster-report	:	1.1.7 (2018)
Percentile statistics	:	20191225.v01 (using entries in the PDB archive December 25th 2019)
Refmac	:	5.8.0158
CCP4	:	7.0.044 (Gargrove)
Ideal geometry (proteins)	:	Engh & Huber (2001)

1 Overall quality at a glance (i)

The following experimental techniques were used to determine the structure:
X-RAY DIFFRACTION

The reported resolution of this entry is 1.56 Å.

Percentile scores (ranging between 0-100) for global validation metrics of the entry are shown in the following graphic. The table shows the number of entries on which the scores are based.

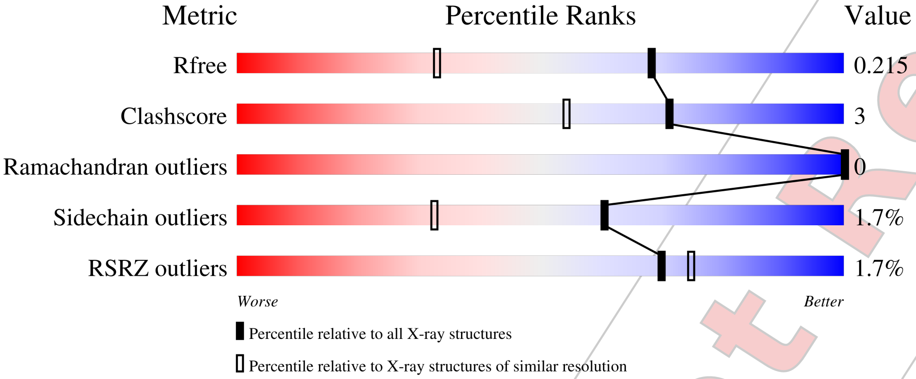

Metric	Whole archive (#Entries)	Similar resolution (#Entries, resolution range(Å))
R_{free}	130704	1483 (1.56-1.56)
Clashscore	141614	1529 (1.56-1.56)
Ramachandran outliers	138981	1498 (1.56-1.56)
Sidechain outliers	138945	1495 (1.56-1.56)
RSRZ outliers	127900	1465 (1.56-1.56)

The table below summarises the geometric issues observed across the polymeric chains and their fit to the electron density. The red, orange, yellow and green segments of the lower bar indicate the fraction of residues that contain outliers for >=3, 2, 1 and 0 types of geometric quality criteria respectively. A grey segment represents the fraction of residues that are not modelled. The numeric value for each fraction is indicated below the corresponding segment, with a dot representing fractions <=5% The upper red bar (where present) indicates the fraction of residues that have poor fit to the electron density. The numeric value is given above the bar.

Mol	Chain	Length	Quality of chain
1	A	518	2%<br>90% 9% •
2	B	2	50% 50%

Ideal geometry (DNA, RNA) : Parkinson et al. (1996)
Validation Pipeline (wwPDB-VP) : 2.34

For Manuscript Review

2 Entry composition (i)

There are 7 unique types of molecules in this entry. The entry contains 4633 atoms, of which 0 are hydrogens and 0 are deuteriums.

In the tables below, the ZeroOcc column contains the number of atoms modelled with zero occupancy, the AltConf column contains the number of residues with at least one atom in alternate conformation and the Trace column contains the number of residues modelled with at most 2 atoms.

- Molecule 1 is a protein called Maltose/maltodextrin-binding periplasmic protein/Induced myeloid leukemia cell differentiation protein Mcl-1 Chimera.

Mol	Chain	Residues	Atoms					ZeroOcc	AltConf	Trace
			Total	C	N	O	S			
1	A	518	4140	2645	696	788	11	4	13	0

There are 7 discrepancies between the modelled and reference sequences:

Chain	Residue	Modelled	Actual	Comment	Reference
A	-196	GLY	-	expression tag	UNP P0AEX9
A	171	GLY	-	linker	UNP P0AEX9
A	172	SER	-	linker	UNP P0AEX9
A	173	GLU	-	linker	UNP P0AEX9
A	194	ALA	LYS	engineered mutation	UNP Q07820
A	197	ALA	LYS	engineered mutation	UNP Q07820
A	201	ALA	ARG	engineered mutation	UNP Q07820

- Molecule 2 is an oligosaccharide called alpha-D-glucopyranose-(1-4)-alpha-D-glucopyranose.

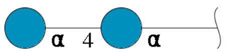

Mol	Chain	Residues	Atoms			ZeroOcc	AltConf	Trace
			Total	C	O			
2	B	2	23	12	11	0	0	0

- Molecule 3 is MAGNESIUM ION (three-letter code: MG) (formula: Mg).

Mol	Chain	Residues	Atoms		ZeroOcc	AltConf
			Total	Mg		
3	A	1	1	1	0	0

- Molecule 4 is (3aM,9S,15R)-4-chloro-3-ethyl-7-{3-[(6-fluoronaphthalen-1-yl)oxy]propyl}

For Manuscript Review

-2-methyl-15-[2-(morpholin-4-yl)ethyl]-2,10,11,12,13,15-hexahydropyrazolo[4',3':9,10][1,6]oxazacycloundecino[8,7,6-hi]indole-8-carboxylic acid (three-letter code: YI7) (formula: $C_{39}H_{44}ClFN_4O_5$) (labeled as "Ligand of Interest" by depositor).

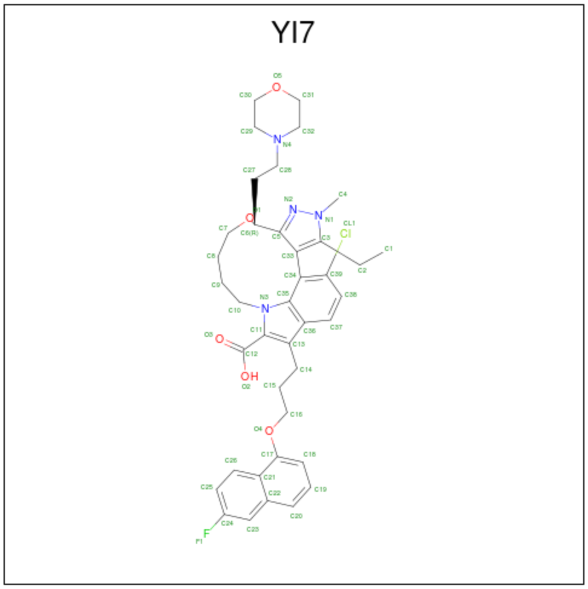

Mol	Chain	Residues	Atoms						ZeroOcc	AltConf
			Total	C	Cl	F	N	O		
4	A	1	50	39	1	1	4	5	0	0

- Molecule 5 is DIMETHYL SULFOXIDE (three-letter code: DMS) (formula: C_2H_6OS).

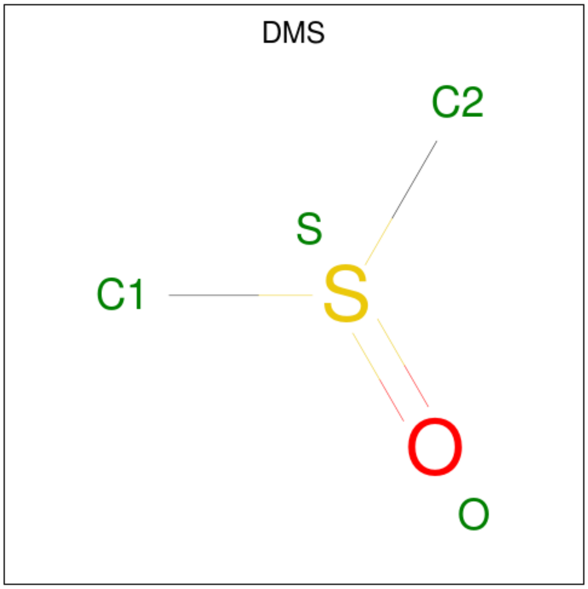

Mol	Chain	Residues	Atoms				ZeroOcc	AltConf
			Total	C	O	S		
5	A	1	4	2	1	1	0	0

For Manuscript Review

- Molecule 6 is TRIETHYLENE GLYCOL (three-letter code: PGE) (formula: $C_6H_{14}O_4$).

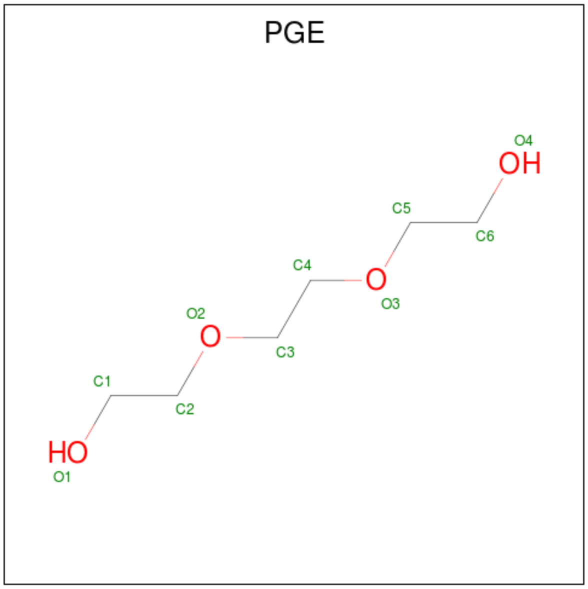

Mol	Chain	Residues	Atoms	ZeroOcc	AltConf
6	A	1	Total C O<br>10 6 4	0	0

- Molecule 7 is water.

Mol	Chain	Residues	Atoms	ZeroOcc	AltConf
7	A	405	Total O<br>405 405	0	0

For Manuscript Review

3 Residue-property plots (i)

These plots are drawn for all protein, RNA, DNA and oligosaccharide chains in the entry. The first graphic for a chain summarises the proportions of the various outlier classes displayed in the second graphic. The second graphic shows the sequence view annotated by issues in geometry and electron density. Residues are color-coded according to the number of geometric quality criteria for which they contain at least one outlier: green = 0, yellow = 1, orange = 2 and red = 3 or more. A red dot above a residue indicates a poor fit to the electron density (RSRZ > 2). Stretches of 2 or more consecutive residues without any outlier are shown as a green connector. Residues present in the sample, but not in the model, are shown in grey.

• Molecule 1: Maltose/maltodextrin-binding periplasmic protein/Induced myeloid leukemia cell differentiation protein Mcl-1 Chimera

Chain A:
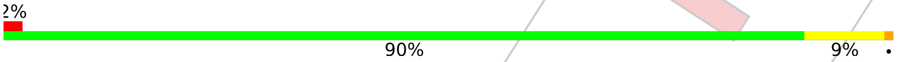

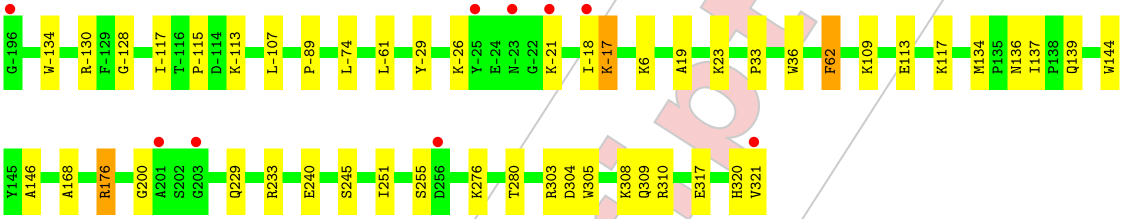

• Molecule 2: alpha-D-glucopyranose-(1-4)-alpha-D-glucopyranose

Chain B:
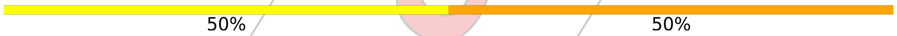

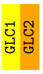

For Manuscript Review

4 Data and refinement statistics (i)

Property	Value	Source
Space group	P 21 21 2	Depositor
Cell constants<br>a, b, c, α, β, γ	98.30Å 136.96Å 38.26Å<br>90.00° 90.00° 90.00°	Depositor
Resolution (Å)	79.86 – 1.56<br>79.86 – 1.56	Depositor<br>EDS
% Data completeness<br>(in resolution range)	99.6 (79.86-1.56)<br>99.6 (79.86-1.56)	Depositor<br>EDS
R_{merge}	0.04	Depositor
R_{sym}	(Not available)	Depositor
$< I/\sigma(I) >$ [1]	2.05 (at 1.56Å)	Xtriage
Refinement program	PHENIX 1.20.1_4487	Depositor
R, R_{free}	0.188 , 0.218<br>0.186 , 0.215	Depositor<br>DCC
R_{free} test set	3735 reflections (5.02%)	wwPDB-VP
Wilson B-factor (Å^2)	25.7	Xtriage
Anisotropy	0.004	Xtriage
Bulk solvent k_{sol}(e/Å^3), B_{sol}(Å^2)	0.32 , 40.2	EDS
L-test for twinning[2]	$< \|L\| > = 0.49$, $< L^2 > = 0.32$	Xtriage
Estimated twinning fraction	No twinning to report.	Xtriage
F_o,F_c correlation	0.97	EDS
Total number of atoms	4633	wwPDB-VP
Average B, all atoms (Å^2)	30.0	wwPDB-VP

Xtriage's analysis on translational NCS is as follows: *The largest off-origin peak in the Patterson function is 4.57% of the height of the origin peak. No significant pseudotranslation is detected.*

[1] Intensities estimated from amplitudes.
[2] Theoretical values of $< |L| >$, $< L^2 >$ for acentric reflections are 0.5, 0.333 respectively for untwinned datasets, and 0.375, 0.2 for perfectly twinned datasets.

For Manuscript Review

5 Model quality (i)

5.1 Standard geometry (i)

Bond lengths and bond angles in the following residue types are not validated in this section: DMS, MG, PGE, GLC, YI7

The Z score for a bond length (or angle) is the number of standard deviations the observed value is removed from the expected value. A bond length (or angle) with $|Z| > 5$ is considered an outlier worth inspection. RMSZ is the root-mean-square of all Z scores of the bond lengths (or angles).

Mol	Chain	Bond lengths		Bond angles	
		RMSZ	#$|Z|$ >5	RMSZ	#$|Z|$ >5
1	A	0.35	0/4240	0.58	0/5741

There are no bond length outliers.

There are no bond angle outliers.

There are no chirality outliers.

There are no planarity outliers.

5.2 Too-close contacts (i)

In the following table, the Non-H and H(model) columns list the number of non-hydrogen atoms and hydrogen atoms in the chain respectively. The H(added) column lists the number of hydrogen atoms added and optimized by MolProbity. The Clashes column lists the number of clashes within the asymmetric unit, whereas Symm-Clashes lists symmetry-related clashes.

Mol	Chain	Non-H	H(model)	H(added)	Clashes	Symm-Clashes
1	A	4140	0	4110	29	0
2	B	23	0	20	1	0
3	A	1	0	0	0	0
4	A	50	0	0	0	0
5	A	4	0	6	0	0
6	A	10	0	14	0	0
7	A	405	0	0	2	0
All	All	4633	0	4150	29	0

The all-atom clashscore is defined as the number of clashes found per 1000 atoms (including hydrogen atoms). The all-atom clashscore for this structure is 3.

All (29) close contacts within the same asymmetric unit are listed below, sorted by their clash magnitude.

For Manuscript Review

Atom-1	Atom-2	Interatomic distance (Å)	Clash overlap (Å)
1:A:-117:ILE:HG22	1:A:-115:PRO:HD3	1.59	0.82
1:A:19:ALA:O	1:A:23:LYS:HD2	1.93	0.69
1:A:109:LYS:O	1:A:113:GLU:HG3	1.99	0.63
1:A:229:GLN:HE21	1:A:233:ARG:HE	1.52	0.58
1:A:-128:GLY:HA3	1:A:136:ASN:O	2.06	0.56
1:A:-17:LYS:HG2	1:A:176:ARG:HG2	1.89	0.54
1:A:305:TRP:O	1:A:309:GLN:HG2	2.08	0.54
1:A:-107:LEU:HD23	1:A:-89:PRO:HG2	1.90	0.54
1:A:-17:LYS:NZ	1:A:200:GLY:HA3	2.23	0.54
1:A:62:PHE:CG	1:A:134:MET:HG2	2.43	0.54
1:A:304:ASP:O	1:A:308:LYS:HG3	2.09	0.52
1:A:310:ARG:HH22	1:A:317[A]:GLU:CD	2.16	0.49
1:A:-134:TRP:CD1	1:A:-130:ARG:HG3	2.48	0.48
1:A:303:ARG:NH1	7:A:509:HOH:O	2.46	0.47
1:A:320:HIS:CE1	1:A:321:VAL:HG13	2.49	0.47
1:A:251:ILE:O	1:A:255:SER:OG	2.25	0.47
1:A:117:LYS:HD3	1:A:117:LYS:HA	1.69	0.47
1:A:33:PRO:HA	1:A:36:TRP:CE2	2.51	0.45
1:A:229:GLN:O	1:A:233:ARG:HG2	2.16	0.45
1:A:6:LYS:NZ	7:A:514:HOH:O	2.50	0.45
1:A:-29:TYR:CZ	1:A:-26:LYS:HE3	2.52	0.44
1:A:229:GLN:HE21	1:A:233:ARG:NE	2.13	0.44
1:A:137:ILE:HD12	1:A:139[B]:GLN:HG2	2.00	0.44
1:A:144:TRP:CD1	2:B:2:GLC:H4	2.54	0.42
1:A:-18:ILE:HD12	1:A:137:ILE:HD13	2.02	0.42
1:A:146:ALA:HB1	1:A:168:ALA:HA	2.02	0.41
1:A:276:LYS:O	1:A:280:THR:HG23	2.21	0.41
1:A:-74:LEU:HD11	1:A:-61:LEU:HD21	2.02	0.41
1:A:-17:LYS:HZ1	1:A:200:GLY:HA3	1.86	0.40

There are no symmetry-related clashes.

5.3 Torsion angles (i)

5.3.1 Protein backbone (i)

In the following table, the Percentiles column shows the percent Ramachandran outliers of the chain as a percentile score with respect to all X-ray entries followed by that with respect to entries of similar resolution.

The Analysed column shows the number of residues for which the backbone conformation was analysed, and the total number of residues.

For Manuscript Review

Mol	Chain	Analysed	Favoured	Allowed	Outliers	Percentiles	
1	A	529/518 (102%)	523 (99%)	6 (1%)	0	100	100

There are no Ramachandran outliers to report.

5.3.2 Protein sidechains (i)

In the following table, the Percentiles column shows the percent sidechain outliers of the chain as a percentile score with respect to all X-ray entries followed by that with respect to entries of similar resolution.

The Analysed column shows the number of residues for which the sidechain conformation was analysed, and the total number of residues.

Mol	Chain	Analysed	Rotameric	Outliers	Percentiles	
1	A	434/421 (103%)	427 (98%)	7 (2%)	62	35

All (7) residues with a non-rotameric sidechain are listed below:

Mol	Chain	Res	Type
1	A	-113	LYS
1	A	-21	LYS
1	A	-17	LYS
1	A	62	PHE
1	A	176	ARG
1	A	240	GLU
1	A	245	SER

Sometimes sidechains can be flipped to improve hydrogen bonding and reduce clashes. All (1) such sidechains are listed below:

Mol	Chain	Res	Type
1	A	229	GLN

5.3.3 RNA (i)

There are no RNA molecules in this entry.

5.4 Non-standard residues in protein, DNA, RNA chains (i)

There are no non-standard protein/DNA/RNA residues in this entry.

For Manuscript Review

5.5 Carbohydrates (i)

2 monosaccharides are modelled in this entry.

In the following table, the Counts columns list the number of bonds (or angles) for which Mogul statistics could be retrieved, the number of bonds (or angles) that are observed in the model and the number of bonds (or angles) that are defined in the Chemical Component Dictionary. The Link column lists molecule types, if any, to which the group is linked. The Z score for a bond length (or angle) is the number of standard deviations the observed value is removed from the expected value. A bond length (or angle) with $|Z| > 2$ is considered an outlier worth inspection. RMSZ is the root-mean-square of all Z scores of the bond lengths (or angles).

Mol	Type	Chain	Res	Link	Bond lengths			Bond angles		
					Counts	RMSZ	$\#\|Z\| > 2$	Counts	RMSZ	$\#\|Z\| > 2$
2	GLC	B	1	2	12,12,12	1.18	2 (16%)	17,17,17	0.94	0
2	GLC	B	2	2	11,11,12	1.25	2 (18%)	15,15,17	0.63	0

In the following table, the Chirals column lists the number of chiral outliers, the number of chiral centers analysed, the number of these observed in the model and the number defined in the Chemical Component Dictionary. Similar counts are reported in the Torsion and Rings columns. '-' means no outliers of that kind were identified.

Mol	Type	Chain	Res	Link	Chirals	Torsions	Rings
2	GLC	B	1	2	-	0/2/22/22	0/1/1/1
2	GLC	B	2	2	-	0/2/19/22	0/1/1/1

All (4) bond length outliers are listed below:

Mol	Chain	Res	Type	Atoms	Z	Observed(Å)	Ideal(Å)
2	B	1	GLC	O3-C3	2.27	1.48	1.43
2	B	2	GLC	O5-C1	2.19	1.47	1.43
2	B	1	GLC	O5-C1	2.05	1.48	1.42
2	B	2	GLC	O3-C3	2.05	1.47	1.43

There are no bond angle outliers.

There are no chirality outliers.

There are no torsion outliers.

There are no ring outliers.

1 monomer is involved in 1 short contact:

Mol	Chain	Res	Type	Clashes	Symm-Clashes
2	B	2	GLC	1	0

For Manuscript Review

The following is a two-dimensional graphical depiction of Mogul quality analysis of bond lengths, bond angles, torsion angles, and ring geometry for oligosaccharide.

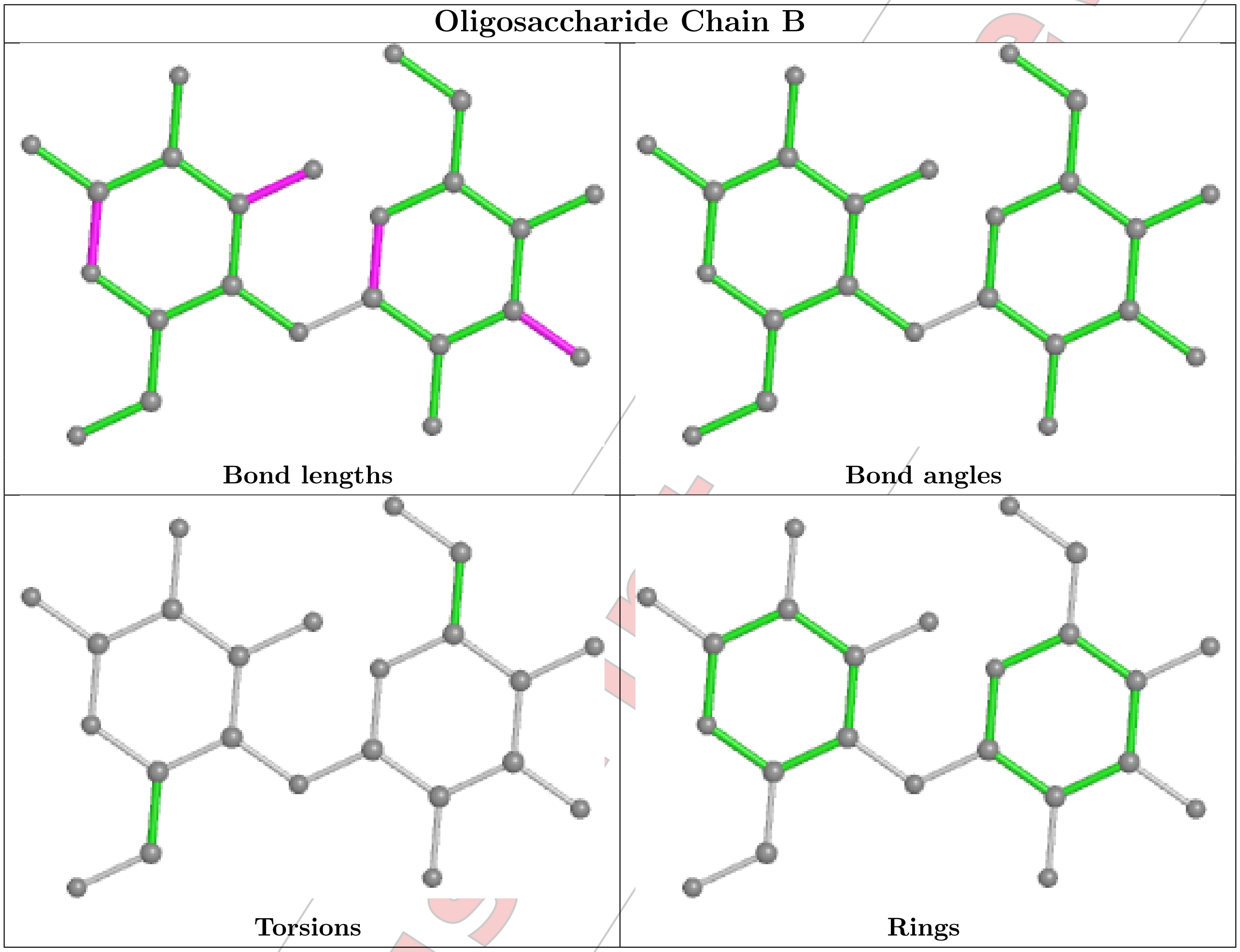

5.6 Ligand geometry (i)

Of 4 ligands modelled in this entry, 1 is monoatomic - leaving 3 for Mogul analysis.

In the following table, the Counts columns list the number of bonds (or angles) for which Mogul statistics could be retrieved, the number of bonds (or angles) that are observed in the model and the number of bonds (or angles) that are defined in the Chemical Component Dictionary. The Link column lists molecule types, if any, to which the group is linked. The Z score for a bond length (or angle) is the number of standard deviations the observed value is removed from the expected value. A bond length (or angle) with $|Z| > 2$ is considered an outlier worth inspection. RMSZ is the root-mean-square of all Z scores of the bond lengths (or angles).

Mol	Type	Chain	Res	Link	Bond lengths			Bond angles		
					Counts	RMSZ	$\#\|Z\| > 2$	Counts	RMSZ	$\#\|Z\| > 2$
5	DMS	A	403	-	3,3,3	0.69	0	3,3,3	0.49	0
6	PGE	A	404	-	9,9,9	0.32	0	8,8,8	0.26	0
4	YI7	A	402	-	49,56,56	1.37	3 (6%)	58,80,80	1.75	12 (20%)

For Manuscript Review

In the following table, the Chirals column lists the number of chiral outliers, the number of chiral centers analysed, the number of these observed in the model and the number defined in the Chemical Component Dictionary. Similar counts are reported in the Torsion and Rings columns. '-' means no outliers of that kind were identified.

Mol	Type	Chain	Res	Link	Chirals	Torsions	Rings
6	PGE	A	404	-	-	3/7/7/7	-
4	YI7	A	402	-	-	8/30/42/42	0/6/7/7

All (3) bond length outliers are listed below:

Mol	Chain	Res	Type	Atoms	Z	Observed(Å)	Ideal(Å)
4	A	402	YI7	C33-C34	6.13	1.54	1.50
4	A	402	YI7	C21-C22	-2.62	1.38	1.43
4	A	402	YI7	C23-C24	2.09	1.39	1.36

All (12) bond angle outliers are listed below:

Mol	Chain	Res	Type	Atoms	Z	Observed(^{o})	Ideal(^{o})
4	A	402	YI7	C5-N2-N1	6.90	110.43	104.48
4	A	402	YI7	C4-N1-C3	-3.92	124.22	128.91
4	A	402	YI7	C33-C34-C39	-3.60	117.31	121.63
4	A	402	YI7	C37-C36-C35	2.60	121.60	116.73
4	A	402	YI7	C25-C24-C23	-2.34	120.65	123.23
4	A	402	YI7	C34-C33-C3	-2.33	123.23	127.67
4	A	402	YI7	C17-C21-C22	2.32	120.93	118.01
4	A	402	YI7	C32-N4-C29	2.19	113.75	108.83
4	A	402	YI7	C3-N1-N2	-2.10	110.86	113.13
4	A	402	YI7	C33-C5-C6	-2.05	126.53	129.87
4	A	402	YI7	C26-C21-C22	2.04	120.53	117.89
4	A	402	YI7	C34-C33-C5	2.01	131.51	127.67

There are no chirality outliers.

All (11) torsion outliers are listed below:

Mol	Chain	Res	Type	Atoms
4	A	402	YI7	C5-C6-O1-C7
4	A	402	YI7	C27-C6-O1-C7
4	A	402	YI7	C13-C11-C12-O2
4	A	402	YI7	C13-C11-C12-O3
4	A	402	YI7	N3-C11-C12-O2
4	A	402	YI7	N3-C11-C12-O3
6	A	404	PGE	O2-C3-C4-O3

Continued on next page...

For Manuscript Review

Continued from previous page...

Mol	Chain	Res	Type	Atoms
4	A	402	YI7	C8-C7-O1-C6
6	A	404	PGE	C4-C3-O2-C2
4	A	402	YI7	O1-C7-C8-C9
6	A	404	PGE	C1-C2-O2-C3

There are no ring outliers.

No monomer is involved in short contacts.

The following is a two-dimensional graphical depiction of Mogul quality analysis of bond lengths, bond angles, torsion angles, and ring geometry for all instances of the Ligand of Interest. In addition, ligands with molecular weight > 250 and outliers as shown on the validation Tables will also be included. For torsion angles, if less then 5% of the Mogul distribution of torsion angles is within 10 degrees of the torsion angle in question, then that torsion angle is considered an outlier. Any bond that is central to one or more torsion angles identified as an outlier by Mogul will be highlighted in the graph. For rings, the root-mean-square deviation (RMSD) between the ring in question and similar rings identified by Mogul is calculated over all ring torsion angles. If the average RMSD is greater than 60 degrees and the minimal RMSD between the ring in question and any Mogul-identified rings is also greater than 60 degrees, then that ring is considered an outlier. The outliers are highlighted in purple. The color gray indicates Mogul did not find sufficient equivalents in the CSD to analyse the geometry.

Ligand YI7 A 402

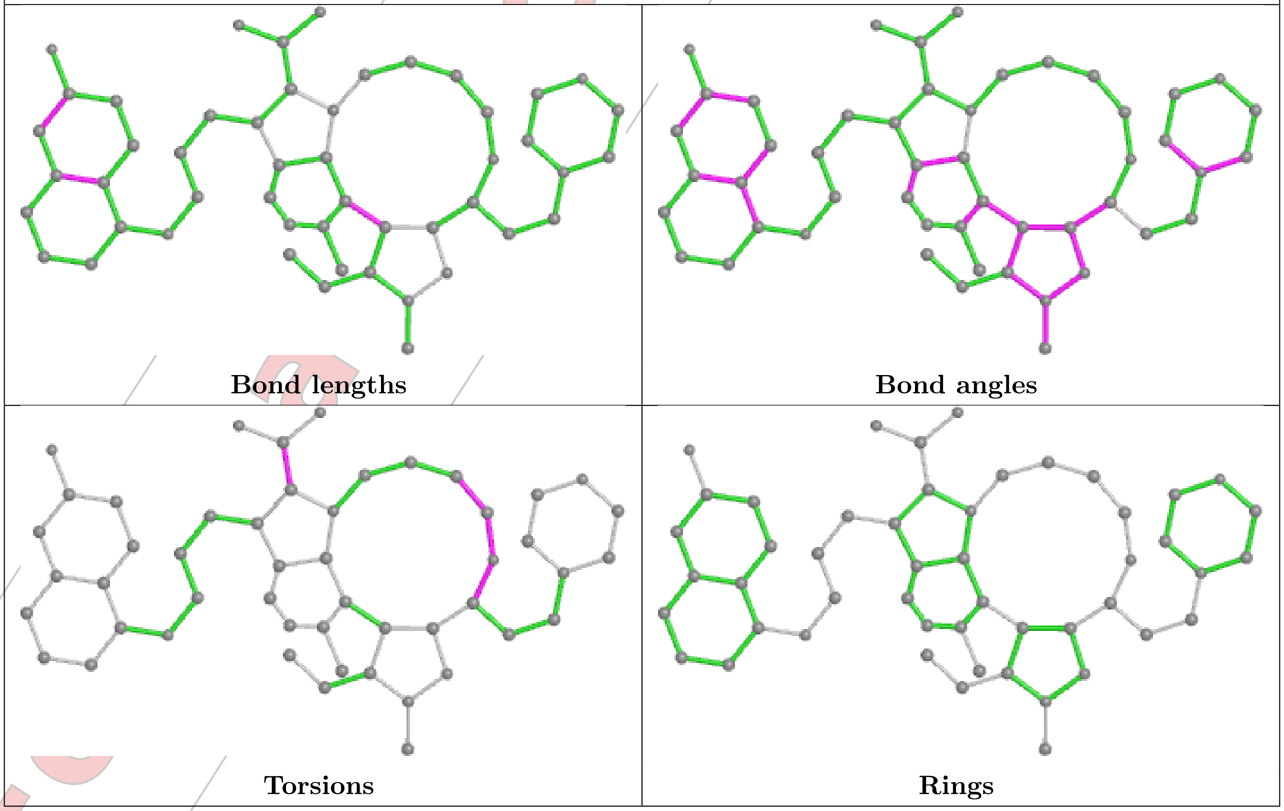

For Manuscript Review

5.7 Other polymers (i)

There are no such residues in this entry.

5.8 Polymer linkage issues (i)

There are no chain breaks in this entry.

For Manuscript Review

6 Fit of model and data (i)

6.1 Protein, DNA and RNA chains (i)

In the following table, the column labelled '#RSRZ> 2' contains the number (and percentage) of RSRZ outliers, followed by percent RSRZ outliers for the chain as percentile scores relative to all X-ray entries and entries of similar resolution. The OWAB column contains the minimum, median, 95th percentile and maximum values of the occupancy-weighted average B-factor per residue. The column labelled 'Q< 0.9' lists the number of (and percentage) of residues with an average occupancy less than 0.9.

Mol	Chain	Analysed	<RSRZ>	#RSRZ>2			OWAB(Å^2)	Q<0.9
1	A	518/518 (100%)	-0.08	9 (1%)	70	75	18, 27, 48, 65	2 (0%)

All (9) RSRZ outliers are listed below:

Mol	Chain	Res	Type	RSRZ
1	A	-23	ASN	4.6
1	A	201	ALA	3.5
1	A	321	VAL	3.4
1	A	-196	GLY	2.8
1	A	-18	ILE	2.8
1	A	-21	LYS	2.5
1	A	256	ASP	2.4
1	A	-25	TYR	2.4
1	A	203	GLY	2.2

6.2 Non-standard residues in protein, DNA, RNA chains (i)

There are no non-standard protein/DNA/RNA residues in this entry.

6.3 Carbohydrates (i)

In the following table, the Atoms column lists the number of modelled atoms in the group and the number defined in the chemical component dictionary. The B-factors column lists the minimum, median, 95th percentile and maximum values of B factors of atoms in the group. The column labelled 'Q< 0.9' lists the number of atoms with occupancy less than 0.9.

Mol	Type	Chain	Res	Atoms	RSCC	RSR	B-factors(Å^2)	Q<0.9
2	GLC	B	1	12/12	0.96	0.06	16,20,26,27	0
2	GLC	B	2	11/12	0.97	0.07	17,18,21,21	0

For Manuscript Review

The following is a graphical depiction of the model fit to experimental electron density for oligosaccharide. Each fit is shown from different orientation to approximate a three-dimensional view.

Electron density around Chain B:

2mF$_o$-DF$_c$ (at 0.7 rmsd) in gray
mF$_o$-DF$_c$ (at 3 rmsd) in purple (negative)
and green (positive)

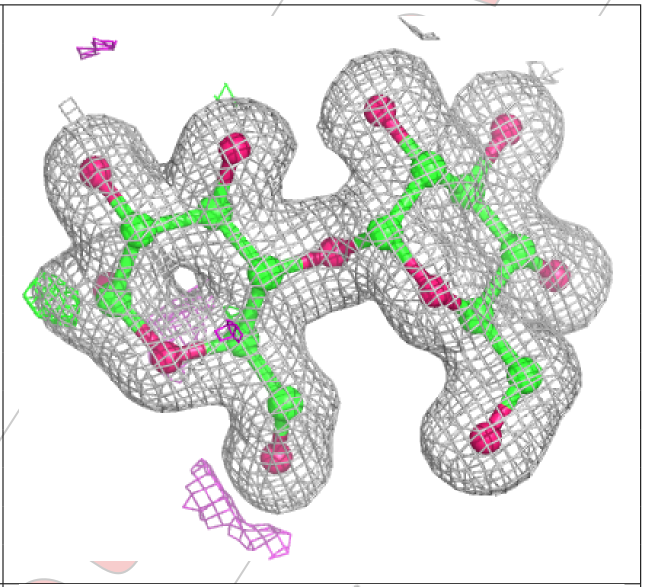

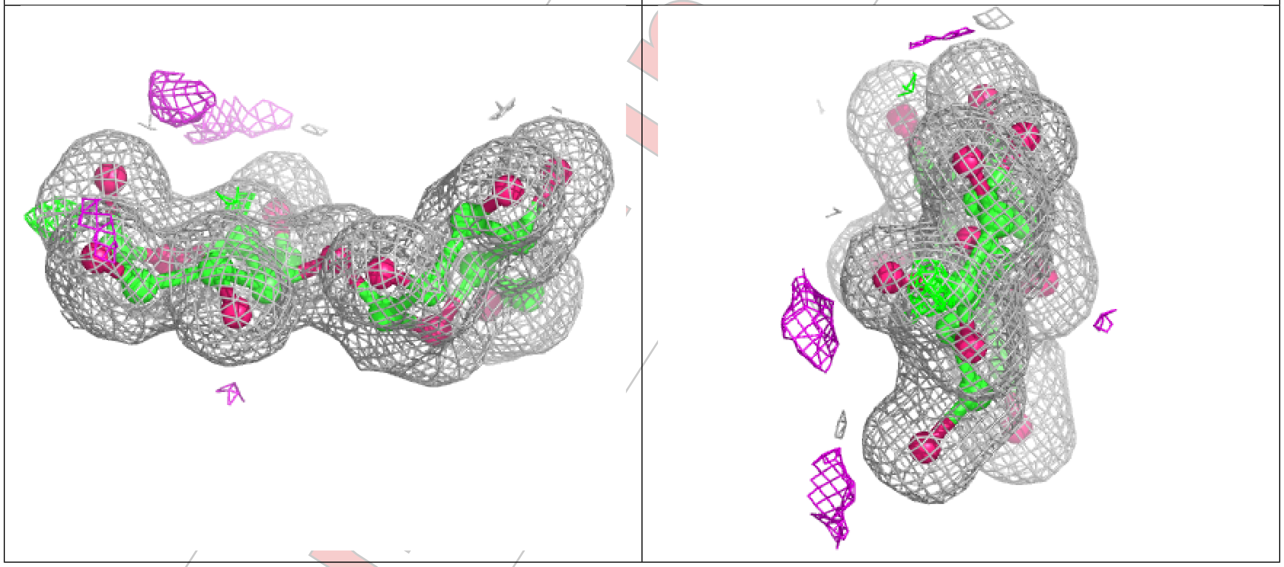

6.4 Ligands ⓘ

In the following table, the Atoms column lists the number of modelled atoms in the group and the number defined in the chemical component dictionary. The B-factors column lists the minimum, median, 95th percentile and maximum values of B factors of atoms in the group. The column labelled 'Q< 0.9' lists the number of atoms with occupancy less than 0.9.

Mol	Type	Chain	Res	Atoms	RSCC	RSR	B-factors(Å^2)	Q<0.9
6	PGE	A	404	10/10	0.79	0.17	43,49,57,58	0
5	DMS	A	403	4/4	0.92	0.29	41,55,58,75	0
4	YI7	A	402	50/50	0.94	0.08	22,30,47,53	0
3	MG	A	401	1/1	0.95	0.20	40,40,40,40	0

For Manuscript Review

The following is a graphical depiction of the model fit to experimental electron density of all instances of the Ligand of Interest. In addition, ligands with molecular weight > 250 and outliers as shown on the geometry validation Tables will also be included. Each fit is shown from different orientation to approximate a three-dimensional view.

Electron density around YI7 A 402:

$2mF_o$-DF_c (at 0.7 rmsd) in gray
mF_o-DF_c (at 3 rmsd) in purple (negative)
and green (positive)

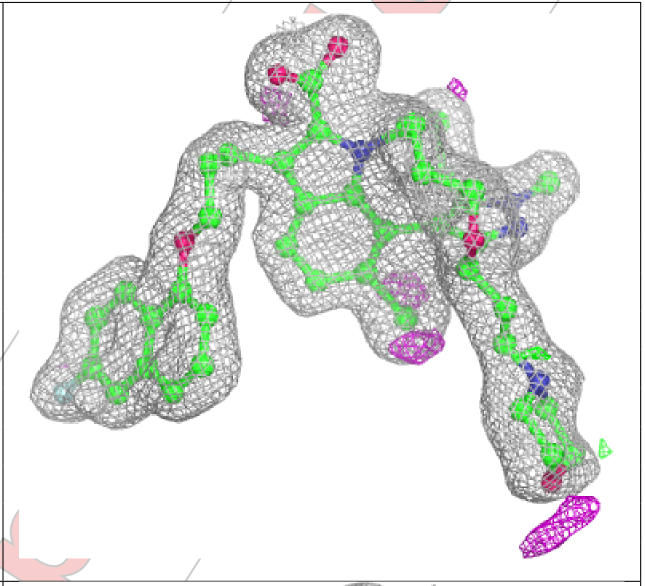

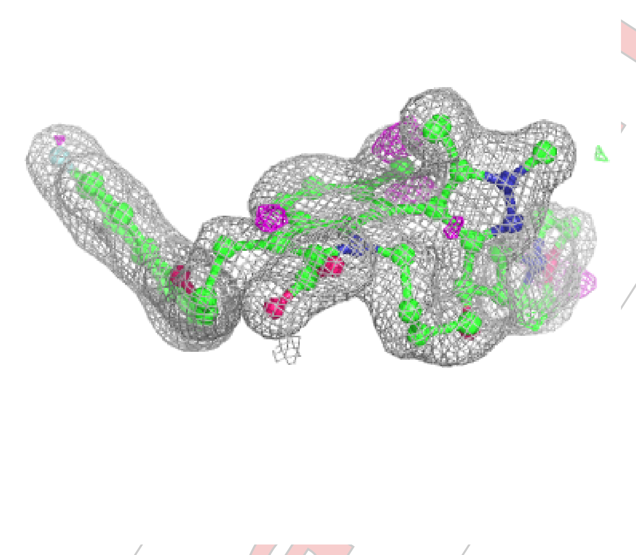

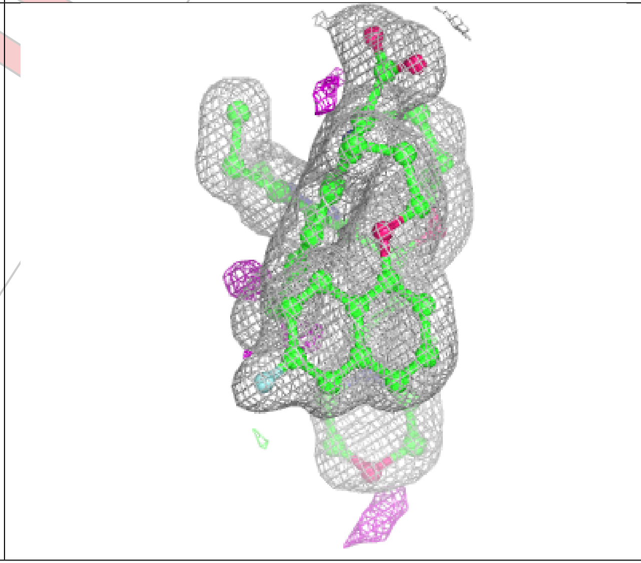

6.5 Other polymers ⓘ

There are no such residues in this entry.

For Manuscript Review
