# Supplementary material for: BRD-810 is a highly selective MCL1 inhibitor with optimized in vivo clearance and robust efficacy in solid and hematological tumor models
Source: Nat Cancer. 2024 Aug 23;5(10):1479–93. doi: 10.1038/s43018-024-00814-0 (PMC11502502; doi:10.1038/s43018-024-00814-0)

Rauh et al, Figure 6

Heart Histopathology, Vehicle group, animal #1005, low magnification

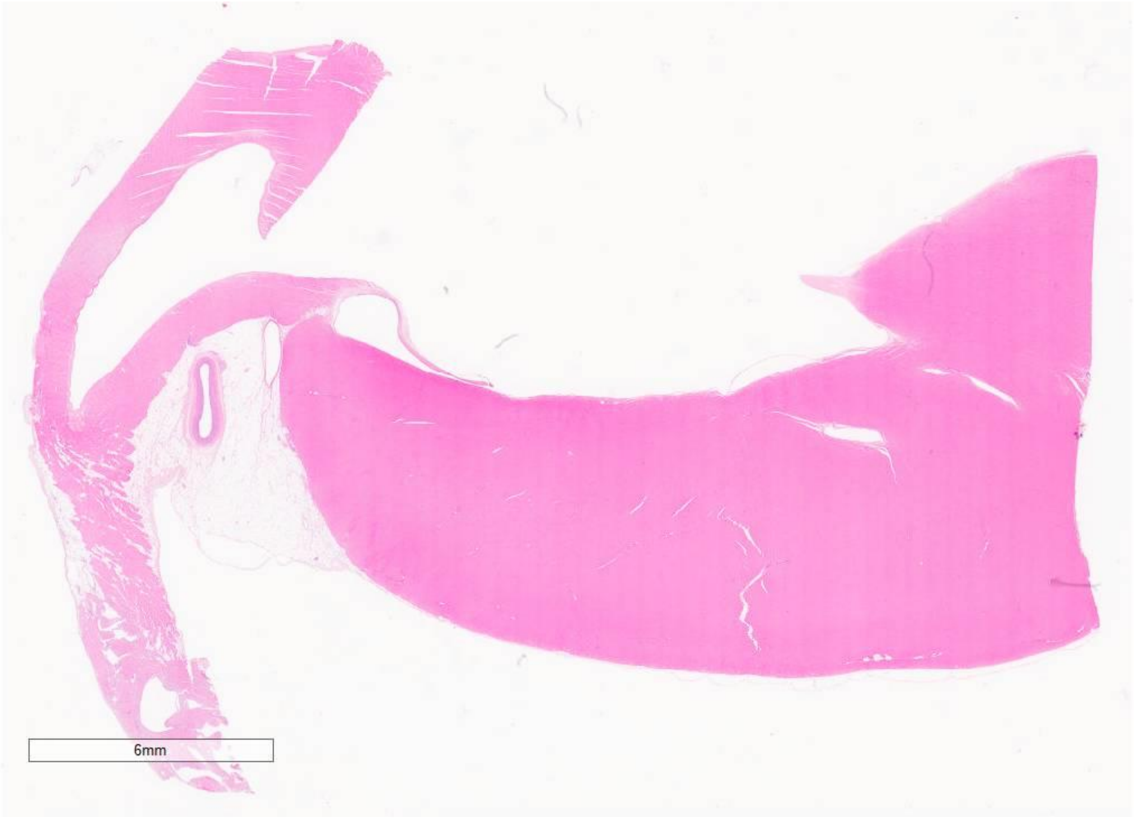

Heart Histopathology, 3mg/kg BRD-810 group, animal #2002, low magnification

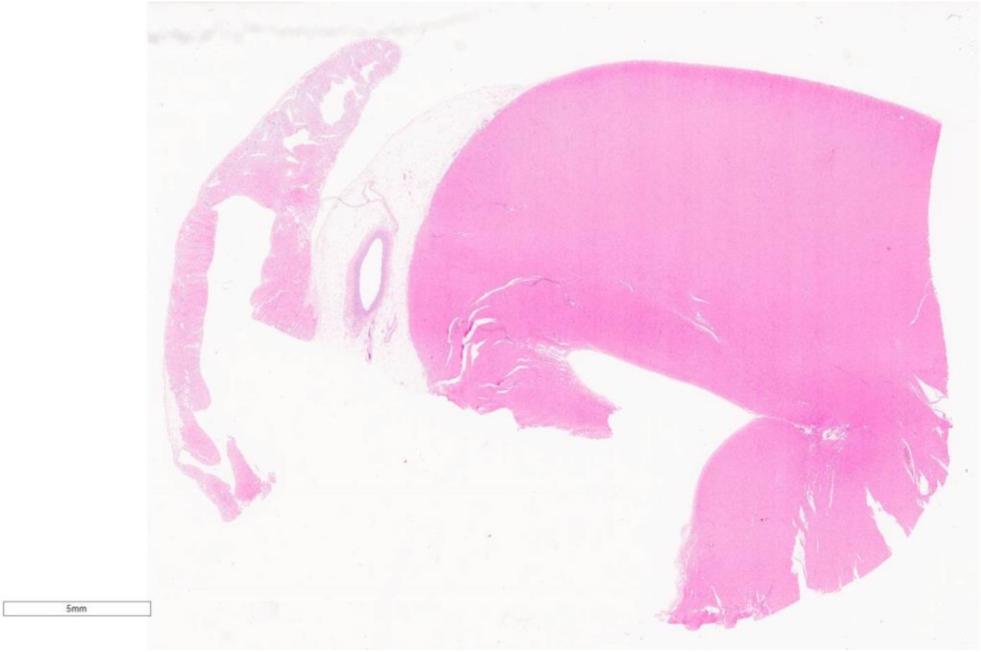

Rauh et al, Figure 6

Heart Histopathology, 8 mg/kg BRD-810 group, animal #3502, low magnification

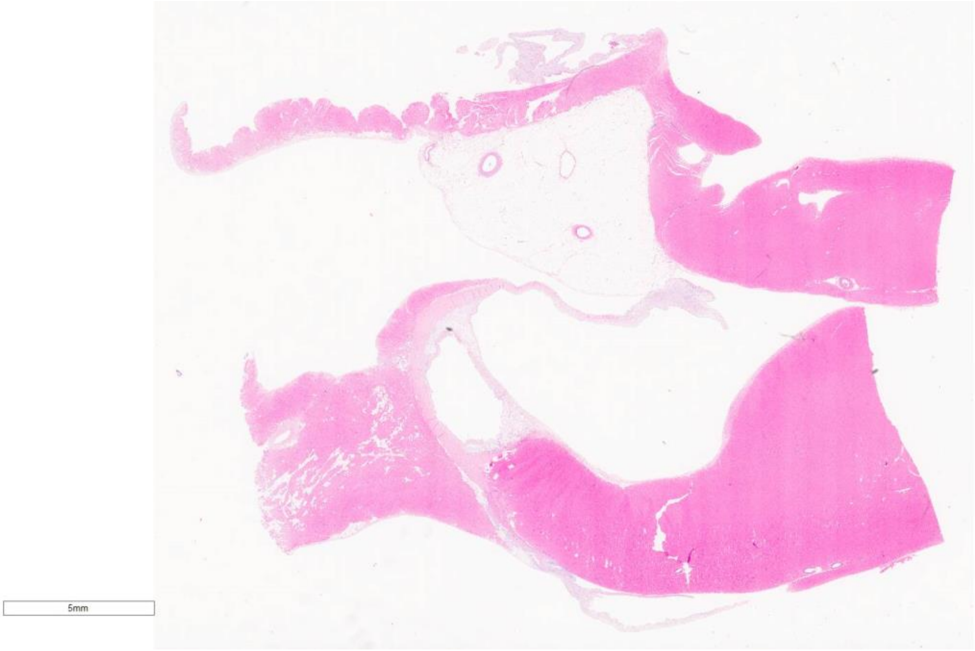

Heart Histopathology, 15 mg/kg BRD-810 group, animal #4005, low magnification

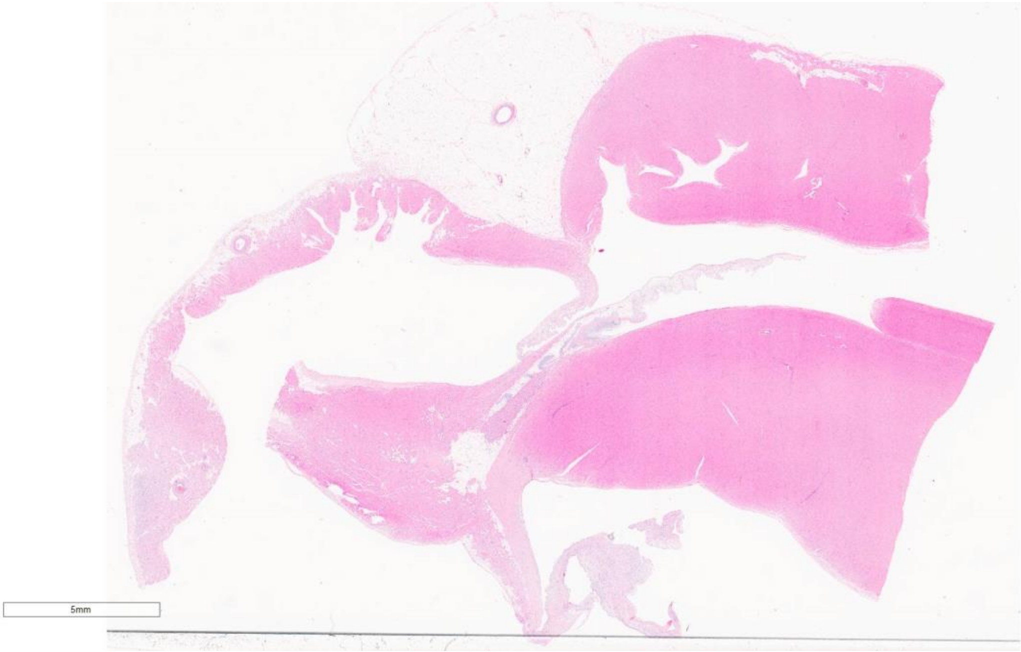

Rauh et al, Figure 6

Heart Histopathology, Vehicle group, animal #1005, low magnification

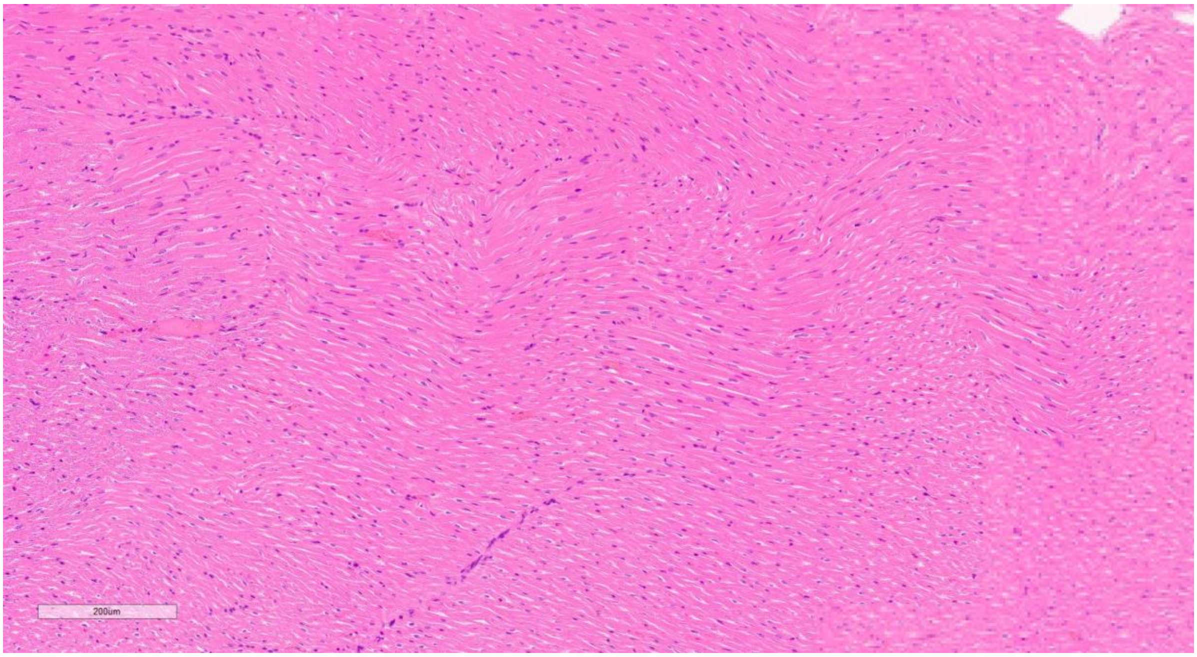

Heart Histopathology, 3mg/kg BRD-810 group, animal #2002, low magnification

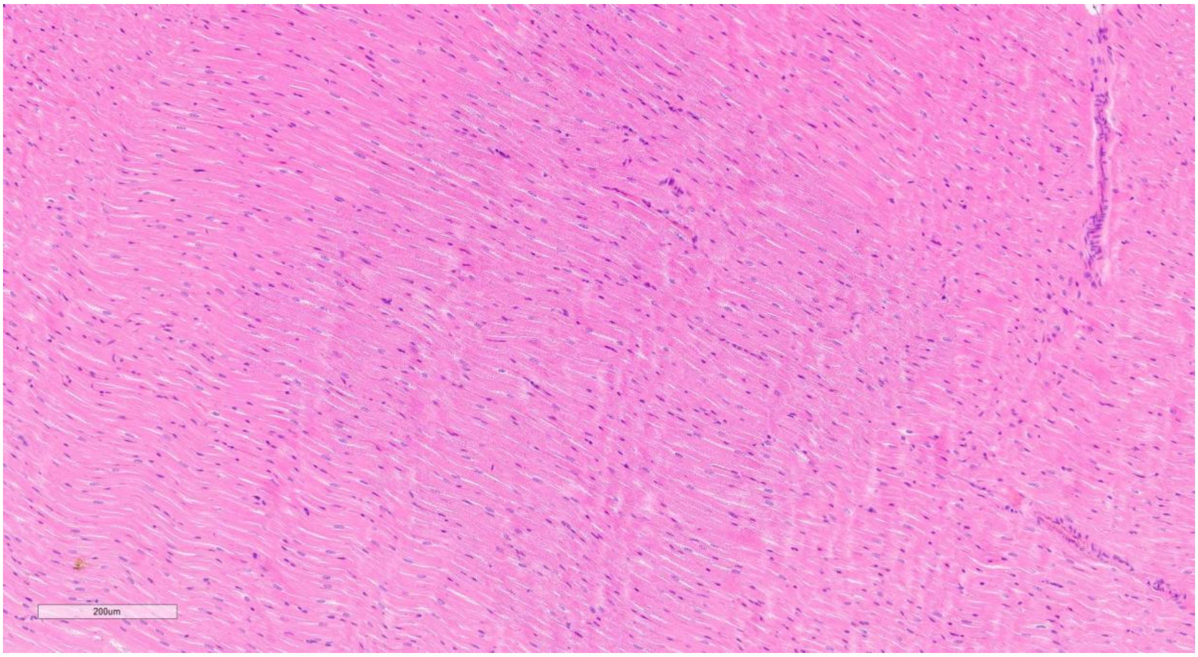

Rauh et al, Figure 6

Heart Histopathology, 8 mg/kg BRD-810 group, animal #3502, low magnification

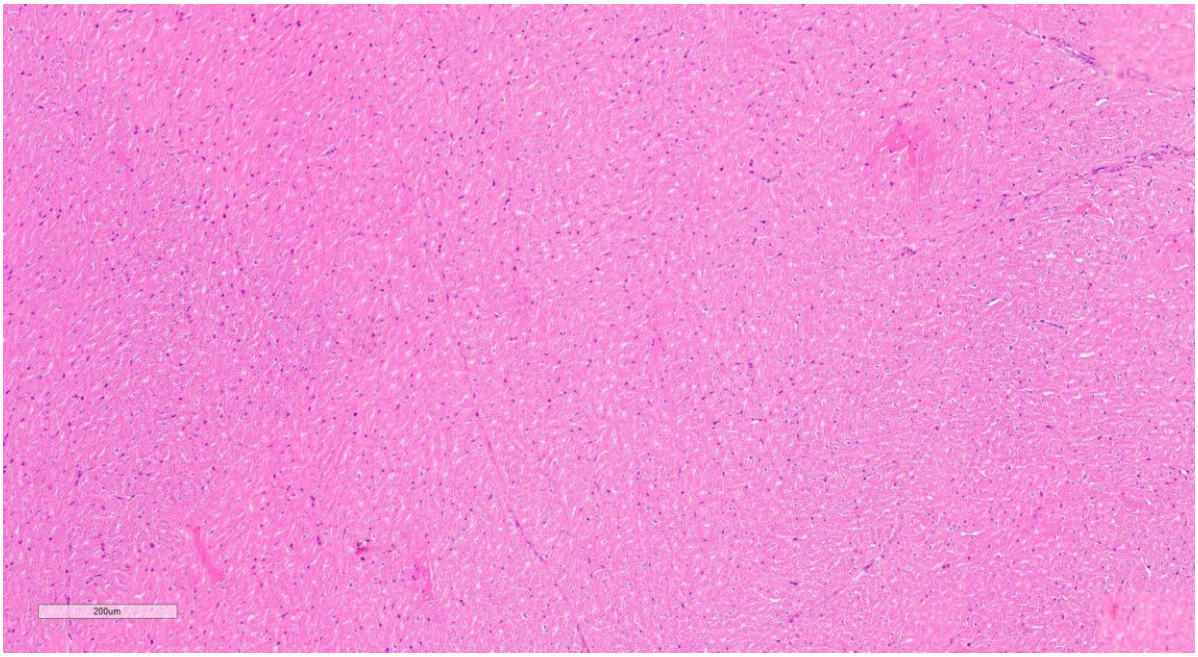

Heart Histopathology, 15 mg/kg BRD-810 group, animal #4005, low magnification

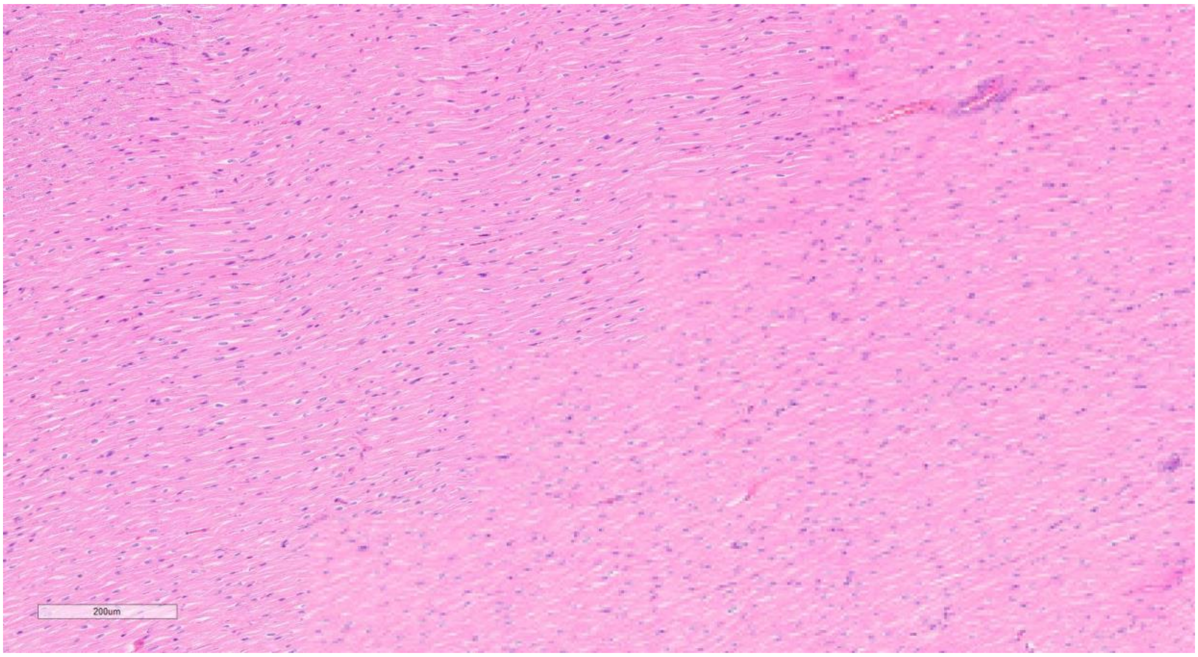

Supplement: Supplementary file 10 — Unprocessed, high-resolution histopathology data. [file 43018_2024_814_MOESM10_ESM.pdf]
